# Remote supervised exercise- and physical activity-based rehabilitation for post-stroke depression, anxiety, and quality of life: a systematic review, evidence map, and exploratory quantitative synthesis

**DOI:** 10.3389/fpsyt.2026.1888180

**Published:** 2026-10-16

**Authors:** Cheng Zhang, Zhou Zhou, Jie Cai, Yuanyuan Li

**Affiliations:** 1 College of Physical Education and Health, Guangxi Normal University, Guilin, China; 2 School of Physical Education, Sichuan Minzu College, Kangding, China

**Keywords:** anxiety, exercise, physical activity, post-stroke depression, quality of life, remote supervision, stroke, telerehabilitation

## Abstract

**Background:**

Depression, anxiety, and reduced quality of life are common after stroke and may interfere with rehabilitation engagement and long-term recovery. Remote supervision can support home-based exercise or physical activity, but the available programs are complex packages that may also include coaching, caregiver involvement, education, and psychological support. We aimed to synthesize randomized evidence on depression, anxiety, and quality of life after remotely supervised exercise- or physical activity-based stroke rehabilitation, accounting for comparator type and co-interventions.

**Methods:**

This systematic review was registered in PROSPERO (CRD420261394314) and reported according to PRISMA 2020. PubMed/MEDLINE, Web of Science, SPORTDiscus, Cochrane Library, Embase, and Scopus were searched from inception to 1 March 2026. Randomized studies of remotely supervised exercise- or physical activity-based rehabilitation after ischemic or hemorrhagic stroke were eligible if depression, anxiety, or quality-of-life outcomes were extractable. Risk of bias was assessed using RoB 2, certainty was evaluated using GRADE, and a co-intervention sensitivity analysis examined the evidence remaining after exclusion of interventions with explicit psychological support.

**Results:**

Six randomized studies involving 483 participants were included. Interventions, comparators, outcome instruments, and study designs were heterogeneous, precluding a primary pooled meta-analysis. The only directly analyzable depression effect against standard care came from Sun et al., a 12-week multidisciplinary telerehabilitation program that included online psychological counseling; the aligned Hedges g was 0.93 (95% CI 0.64 to 1.22), conventionally large, but certainty was very low. After this trial was excluded, no directly analyzable depression effect against standard care remained. Active-vs-active depression or distress findings did not establish superiority. Quality-of-life signals varied across instruments and comparators; no separable patient anxiety effect was available.

**Conclusion:**

The included evidence concerns complex remote rehabilitation packages rather than exercise alone. A possible depression benefit is supported principally by one trial with a psychological-support co-intervention and is therefore fragile. Remote programs should not be regarded as stand-alone treatments for post-stroke depression or anxiety. Confirmatory trials should recruit or stratify participants by baseline psychiatric symptoms, prespecify psychiatric outcomes, and separate exercise from psychological and implementation components.

**Systematic review registration:**

https://www.crd.york.ac.uk/PROSPERO/view/CRD420261394314, identifier CRD420261394314.

## Introduction

Stroke remains a leading cause of adult disability, long-term dependence and participation restriction worldwide (1). Contemporary rehabilitation guidance emphasizes early multidisciplinary care, long-term physical activity and exercise prescription, and transition from institution-based rehabilitation to supported community living (2, 3). For many survivors, however, recovery is shaped not only by motor impairment but also by psychological distress, fatigue, social isolation and reduced participation.

Post-stroke depression is among the most frequent neuropsychiatric complications after stroke (4, 5). A scientific statement from the American Heart Association/American Stroke Association and large systematic reviews report that depressive symptoms are common and are associated with poorer functional recovery, reduced quality of life, and adverse long-term outcomes (4, 5). Anxiety is also prevalent after stroke, although it is less consistently measured and is often combined with depression or general psychological distress in rehabilitation trials (6). These psychiatric outcomes matter because they may reduce motivation, adherence, and confidence in rehabilitation, thereby affecting both emotional recovery and functional participation.

Quality of life and health-related quality of life are central patient-reported outcomes in stroke recovery because they integrate physical function, emotion, social participation and perceived recovery. Stroke-specific instruments such as the Stroke-Specific Quality of Life Scale and the Stroke Impact Scale were developed to capture domains that are meaningful to stroke survivors (7, 8). For a psychiatry and rehabilitation audience, these measures are not secondary conveniences but clinically important endpoints that reflect whether recovery translates into lived well-being.

Remote supervision was treated in this review as a delivery mode, not as a biologically distinct form of exercise. It can extend monitoring and feedback into the home and may reduce travel or continuity barriers, but the included trials did not directly measure reach, equity, or access. Broader telerehabilitation evidence indicates that selected functional outcomes can be comparable with in-clinic therapy (9, 10), whereas physical fitness training after stroke improves mobility and fitness more consistently than psychiatric endpoints (11). We focused on remote delivery because its implementation components and comparator structures may modify observed mental-health effects and therefore require separate interpretation from face-to-face supervised exercise.

Exercise and physical activity could influence mood and quality of life through several interacting pathways. Broader mechanistic literature proposes that exercise-related neurotrophic signaling supports neural plasticity, while changes in inflammatory and stress-response systems may influence emotional regulation (12). Graded activity can also provide achievable goals and mastery experiences, supporting self-efficacy; for anxiety, repeated experience of tolerable exertional sensations may reduce fear of those sensations and activity avoidance (13). Improvements in physical capacity may facilitate everyday activities and participation, which could improve perceived well-being independently of a change in psychiatric symptoms (11, 12). These are plausible pathways, not mechanisms established by the stroke trials reviewed here. Remote supervision may support them through feedback and therapeutic contact, rather than constituting a distinct biological treatment.

However, digital and home-based programs are complex interventions. They may combine exercise, education, self-monitoring, wearable feedback, therapist coaching, caregiver support, social contact, and psychological support. Stroke recovery research has called for clearer definitions and reproducible reporting, and complex-intervention guidance emphasizes identifying which components are being evaluated and under what contextual conditions (14–16). A mental-health-focused synthesis must therefore distinguish direct exercise content from the broader effects of remote supervision, behavioral coaching, psychological support, and implementation assistance.

Recent syntheses address related but distinct questions. A 2025 review reported improvements in depressive symptoms across exercise interventions after stroke, without isolating remote supervision (17). A 2024 review of home-based exercise found very-low-certainty evidence for post-stroke depression; home-based delivery does not itself establish remote supervision (18). A 2024 telerehabilitation review found no clear between-group difference in its pooled Stroke Impact Scale analysis (19). These findings do not establish whether mental-health effects differ by remote intervention content, comparator, or psychological co-intervention. A focused synthesis is therefore needed to distinguish psychiatric measures from quality-of-life domains and to identify which randomized contrasts support each conclusion.

We aimed to synthesize randomized evidence on depression, anxiety, and quality of life after remotely supervised exercise- or physical activity-based stroke rehabilitation, accounting for comparator type and co-interventions. The review also summarizes activities of daily living, motor function, adherence, and safety as contextual, exploratory rehabilitation outcomes; distinguishes exercise content from psychological, coaching, and implementation co-interventions; and documents when statistical pooling, funnel plots, network meta-analysis, or dose-response analysis were not methodologically defensible.

## Methods

### Study design, registration and reporting standards

This systematic review and exploratory quantitative synthesis was registered in PROSPERO (CRD420261394314). The review was reported according to PRISMA 2020 and was planned in accordance with the Cochrane Handbook for Systematic Reviews of Interventions. Where statistical pooling was not appropriate, narrative synthesis followed SWiM principles, and synthesis decisions were documented in source-traceable datasets to improve reproducibility (20–22).

### Eligibility criteria

Population: adults aged 18 years or older with ischemic or hemorrhagic stroke were eligible. Mixed populations were included only when data for participants with stroke were extractable separately.

Intervention: eligible interventions paired active exercise or physical-activity rehabilitation with remote supervision, monitoring, feedback, or progression delivered by video, telephone, app, web, mHealth, wearable, or a connected rehabilitation device. Home programs without a remote supervisory component and digital interventions without active exercise or physical activity were ineligible.

Comparators: eligible randomized comparisons were usual or standard care, waiting list or no intervention, health education, unsupervised home exercise, face-to-face or conventional rehabilitation, dose-matched home exercise, or another active remote strategy. Uncontrolled studies were ineligible.

Outcomes: the primary outcomes were depression, anxiety, and quality of life or health-related quality of life measured using validated instruments. Activities of daily living, motor function, adherence, safety, and satisfaction were secondary, contextual outcomes and were not treated as substitutes for psychiatric outcomes. Outcomes were eligible whether designated primary or secondary in the source trial, provided that extractable post-intervention, change-score, adjusted, or model-based data were available. Global quality-of-life measures and individual Stroke Impact Scale domains were distinguished; functional domains were interpreted contextually rather than as psychiatric outcomes.

Study design: randomized controlled trials, cluster randomized trials, randomized crossover trials, pilot or feasibility randomized trials, and sequential multiple assignment randomized trials were eligible. Protocols, reviews, meta-analyses, conference abstracts without full data, non-randomized studies, and non-human studies were excluded.

### Information sources and search strategy

PubMed, Web of Science, SPORTDiscus, Cochrane Library, Embase and Scopus were searched from inception to 1 March 2026. The recorded strategies combined stroke, remote rehabilitation, exercise or physical rehabilitation, depression/anxiety/quality of life, and randomized trials. The mandatory outcome block was used to focus retrieval on the review question, not because secondary outcomes were ineligible. Because this restriction could theoretically reduce sensitivity when psychiatric or quality-of-life outcomes were secondary, poorly indexed, or absent from searchable fields, we conducted a supplementary higher-sensitivity verification search without requiring depression, anxiety, or quality-of-life terms. Records identified through this supplementary search were screened using the same eligibility criteria as the original review, and no additional eligible randomized study was identified. The complete database-specific strategies, platforms, original search date, and record counts reported in **Supplementary Table S1** correspond to the original outcome-filtered searches that generated the records in the PRISMA flow diagram; these original counts were therefore retained unchanged. The supplementary search was used specifically to verify the sensitivity of the original outcome restriction and did not alter the final included-study set.

### Selection process and data extraction

Two reviewers independently screened titles and abstracts. Potentially relevant reports were assessed at full-text level by two reviewers independently. Disagreements were resolved by discussion; unresolved disagreements were adjudicated by a third reviewer. Reasons for exclusion at full-text level were recorded and summarized in the PRISMA flow diagram. Trials reporting only physical-function outcomes without eligible depression, anxiety, or quality-of-life information were not included; SIS domain results were not equated with global quality of life.

Two reviewers independently extracted study characteristics, participant eligibility, stroke stage, baseline psychiatric eligibility and symptom characteristics, intervention content, mental-health and implementation co-interventions, supervision mode, technology platform, comparator details, outcome instruments, time points, quantitative results, adherence, safety, and funding or conflict-of-interest information using a standardized extraction form. Extracted quantitative data were checked against the source reports. Complete outcome-level extraction, including result type, direction alignment, synthesis role, and source location, is provided in **Supplementary Table S2**.

### Risk of bias and certainty of evidence

Risk of bias for randomized studies was assessed using the Cochrane RoB 2 framework across five domains: randomization process, deviations from intended interventions, missing outcome data, measurement of the outcome, and selection of the reported result (23). RoB 2 was applied to one representative review-relevant result at the principal post-intervention time point for each trial; these judgments were not assumed to apply to all outcomes reported by the same trial. Feasibility status and comparator choice were treated as applicability or precision issues, not as risk-of-bias domains. Judgments were recorded as low risk, some concerns, or high risk. Representative-result domain judgments and reasons are provided in **Supplementary Table S3**.

The certainty of evidence for the main outcome bodies was assessed using GRADE (24). Evidence from randomized trials started as high certainty and was downgraded for risk of bias, inconsistency, indirectness, imprecision and publication bias. Certainty was rated as high, moderate, low or very low where an interpretable outcome-specific evidence body was available. Anxiety was not assigned a GRADE rating because no separable patient anxiety effect was available; HADS total was retained as combined distress rather than an anxiety-specific estimate. Descriptive evidence sets and the psychological-support sensitivity decision are documented in **Supplementary Table S4**, potential heterogeneity variables in **Supplementary Table S5**, and outcome-level certainty judgments in **Supplementary Table S6**.

### Data synthesis and statistical transparency

The primary synthesis grouped studies by outcome domain, comparator type, and intervention-component profile. Usual or standard care, active conventional rehabilitation, and active remote comparators were considered separately. Standalone depression measures, combined distress measures, global quality-of-life measures, and individual Stroke Impact Scale domains were distinguished; caregiver outcomes were not treated as patient psychiatric outcomes. Intervention profiles distinguished activity-use promotion, structured exercise, caregiver-mediated practice, and multidisciplinary rehabilitation with psychological support. For continuous outcomes reported as means and standard deviations, Hedges g and 95% confidence intervals were calculated using standard inverse-variance methods and directionally aligned so that positive values favored the remote intervention package (25). For lower-is-better scales, signs were reversed before display. Direction alignment harmonized favorable direction only; it did not make different constructs, instruments, metric types, time points, or comparator structures clinically or statistically commensurable. When group-specific standard deviations or covariance-adjusted between-group estimates were unavailable, source-reported within-group changes, interaction tests, or group-specific adjusted marginal means were retained narratively without reconstructing between-group confidence intervals. Medians, interquartile ranges, adjusted model estimates, and SMART model estimates were extracted but not converted or pooled when doing so would add unsupported precision (26).

To aid interpretation, standardized effects were described using conventional approximate thresholds of 0.2, 0.5, and 0.8 as small, moderate, and large, respectively; these labels were used only as statistical descriptors and not as thresholds for clinical importance (27). Random-effects meta-analysis was prespecified only when at least two clinically comparable studies reported the same construct in a sufficiently comparable contrast with analyzable data. No primary outcome met this threshold, so pooled summary effects were not computed. A co-intervention sensitivity analysis excluded trials with explicit psychological support and assessed whether a directly analyzable depression contrast against standard care remained. Because no pooled depression estimate existed, the sensitivity result was expressed as a change in the available evidence set rather than as a recalculated summary effect. The PRISMA box labeled quantitative synthesis therefore denotes quantitative extraction and display rather than a pooled primary meta-analysis.

Funnel plots and formal small-study-effect tests were not produced because fewer than 10 studies were available in any comparable synthesis. Network meta-analysis and dose-response analysis were judged inapplicable because the evidence did not form a connected comparable treatment network and because dose metrics were heterogeneous across interventions. These decisions were made before interpretation of effect direction and are documented in the supplementary data package.

## Results

### Study selection

The database searches identified 2,457 records: PubMed (n=454), Web of Science (n=691), Cochrane Library (n=242), Scopus (n=321), SPORTDiscus (n=105) and Embase (n=644). After removal of 1,805 duplicates, 652 records were screened and 425 were excluded at title/abstract stage. A total of 227 full-text records were assessed, 221 were excluded and six randomized studies were retained for the final qualitative and quantitative evidence synthesis: Fusari et al. (28), Grau-Pellicer et al. (29), Linder et al. (30), Sun et al. (31), van den Berg et al. (32) and Whiston et al. (33) (**Figure 1**). One additional randomized virtual upper-limb activity trial was checked as a borderline record but was not counted in the main synthesis because follow-up depression, anxiety or global quality-of-life data were not extractable (34).

**Figure 1:**
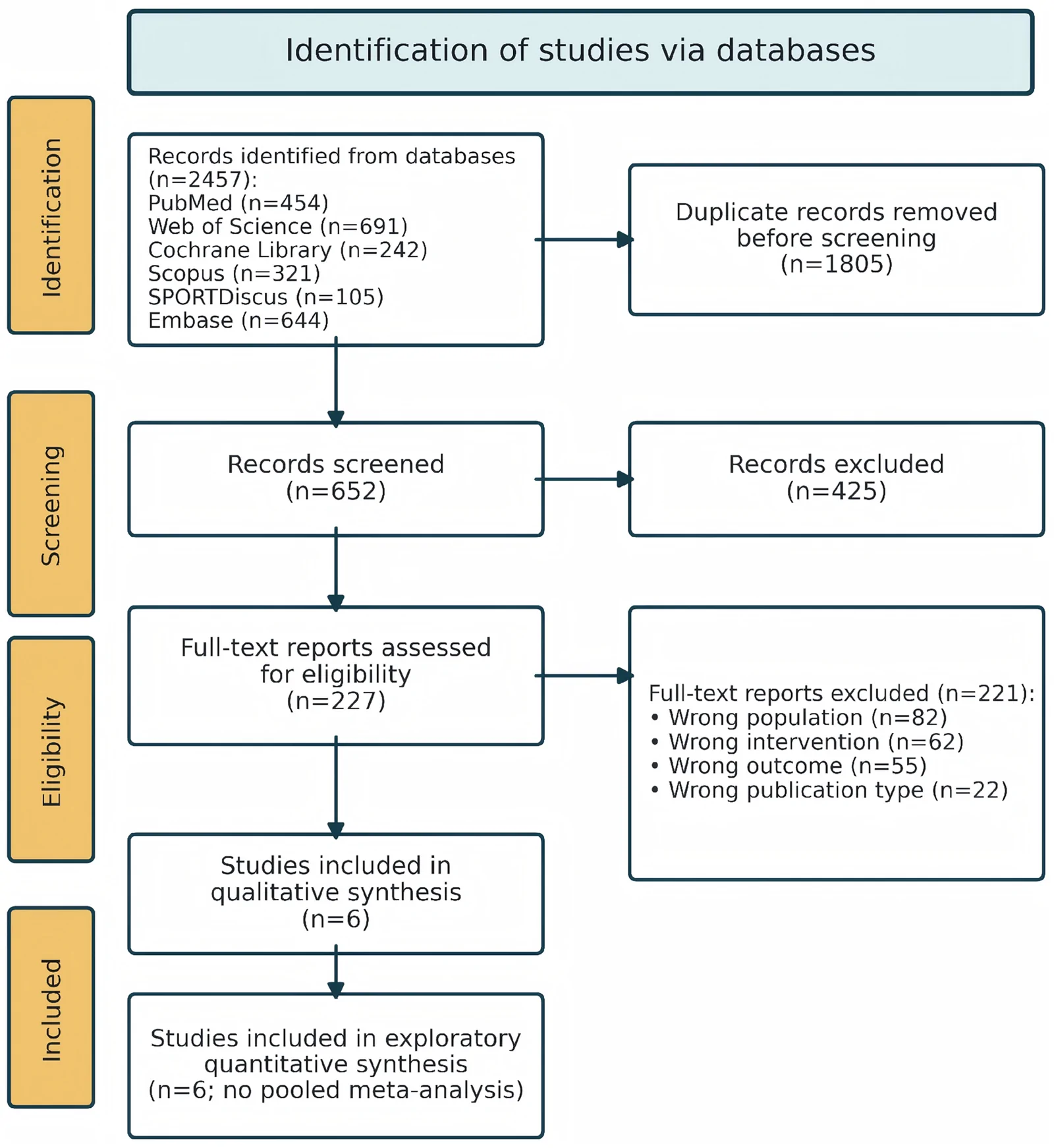
PRISMA 2020 flow diagram. The figure shows database identification, duplicate removal, title/abstract screening, full-text assessment, reasons for full-text exclusion, and final inclusion. Six studies contributed to qualitative synthesis and exploratory quantitative display; no pooled meta-analysis was performed.

### Study characteristics

The six main included studies were published between 2015 and 2026 and enrolled 483 randomized participants. Trials were conducted in the United Kingdom, Spain, the United States, China, Australia, and Ireland. Sample sizes ranged from 30 to 200 participants. Stroke stages varied from early post-discharge acute ischemic stroke to chronic community-dwelling stroke. Designs included two-arm RCTs, pilot or feasibility RCTs, a proof-of-concept RCT, and a feasibility SMART design (**Table 1**). The aggregated overview separates study setting, design, stroke-type reporting, and intervention duration; report titles, objectives, and individual study details are provided in **Table 2**. No trial recruited participants on the basis of a confirmed depressive or anxiety disorder. Sun et al. reported mean baseline HAMD scores of 17.5 and 17.3, Linder et al. reported CES-D scores above 16 in approximately 27% of completers in each arm, and van den Berg et al. initially used a HADS depression screening restriction that was later removed. Patient and caregiver HADS measures were collected in that proof-of-concept trial, but the accessible main report did not provide a separable patient anxiety effect; psychiatric baseline information was limited in the other trials (**Supplementary Table S7**).

**Table 1:** Overview of the six included randomized studies.

Characteristic	Category	Studies, n	Study identification/clarification
Evidence base	Randomized studies/participants	6	483 randomized participants; trial sizes 30-200; publications 2015-2026.
Country	United Kingdom, Spain, United States, China, Australia, Ireland	1 each	Fusari; Grau-Pellicer; Linder; Sun; van den Berg; Whiston, respectively.
Setting/care pathway	Home or community rehabilitation	2	Linder; Whiston.
	Acute/community services or early discharge transition	3	Fusari; Sun; van den Berg.
	Blended supervised outpatient and home activity	1	Grau-Pellicer.
Study design	Parallel-group RCT (not designated pilot/feasibility)	2	Linder; Sun.
	Parallel-group pilot/feasibility RCT	2	Fusari; Grau-Pellicer.
	Proof-of-concept RCT	1	van den Berg.
	Feasibility SMART	1	Whiston.
Stroke type	Ischemic only	1	Sun.
	Ischemic and hemorrhagic stroke represented	3	Fusari; Linder; Whiston.
	Stroke subtype not separately characterized in the available extraction	2	Grau-Pellicer; van den Berg. Not assumed to be ischemic-only or mixed.
	Hemorrhagic-only study identified	0	No study identified as hemorrhagic-only.
Intervention duration	8 weeks	3	Grau-Pellicer; Linder; van den Berg.
	12 weeks	3	Fusari; Sun; Whiston.
Psychiatric recruitment	Confirmed depression/anxiety diagnosis required	0	No included study used a confirmed psychiatric diagnosis as an entry requirement.

Counts describe studies, not participants. Setting and design categories are mutually exclusive descriptive groupings for this table; all studies were randomized. Stroke-type categories retain uncertainty rather than infer subtype from eligibility alone. Treatment duration is distinct from outcome assessment. RCT, randomized controlled trial; SMART, sequential multiple assignment randomized trial.

**Table 2:** Consolidated study-level extraction: report identity, objectives, intervention, outcomes, and synthesis role.

A. Report identity and study characteristics.					
Study/country	Report title	Study objective	Design and setting	Population/stroke stage	Randomized N; treatment period
Fusari 2025 (28) United Kingdom	UPBEAT Trial: a randomised feasibility evaluation of a digital system for UPper limB rEhabilitation After sTroke	Evaluate the feasibility of implementing OnTrack within NHS stroke services and inform a future definitive RCT.	Two-arm parallel randomized feasibility trial across acute and community NHS services.	Arm weakness within 12 months after stroke; ischemic and hemorrhagic stroke (25 and 5 participants, respectively).	30 (15/15); 12 weeks
Grau-Pellicer 2020 (29) Spain	Impact of mHealth technology on adherence to healthy PA after stroke: a randomized study	Evaluate an mHealth-supported physical-activity program for adherence to healthy activity after stroke, with functional and quality-of-life outcomes.	Small unblinded pilot RCT; blended outpatient and home/community activity.	Chronic stroke; FAC >=3, BI >=45, MMSE >24. Stroke subtype not separately characterized in the available extraction.	41 (24/17); 8 weeks; outcomes at 3 months
Linder 2015 (30) United States	Improving quality of life and depression after stroke through telerehabilitation	Compare quality of life and depressive symptoms after robot-assisted home rehabilitation versus a home exercise program.	Multicenter randomized trial; home-based rehabilitation with telephone monitoring in both arms.	Ischemic or hemorrhagic stroke within 6 months; upper-limb limitation and limited access to formal rehabilitation.	99 (51/48); 8 weeks
Sun 2025 (31) China	A randomized controlled trial of telerehabilitation intervention for acute ischemic stroke patients post-discharge	Evaluate a post-discharge telerehabilitation package against standard care for recovery, depression, and quality of life.	RCT with blinded outcome assessment and reported intention-to-treat analysis; early post-discharge care.	Acute ischemic stroke; age 18–75 years; BI <=95; no severe cognitive impairment.	200 (100/100); 12 weeks
van den Berg 2016 (32) Australia	Early supported discharge by caregiver-mediated exercises and e-health support after stroke: a proof-of-concept trial	Test the feasibility and preliminary effects of caregiver-mediated exercises with e-health support during the early discharge pathway.	Pragmatic proof-of-concept RCT; early rehabilitation and transition home.	Stroke 24 hours to 3 months earlier; mobility limitation and an available caregiver. Subtype not separately characterized in the available extraction.	63 (31/32); 8 weeks; assessments at 8 and 12 weeks
Whiston 2026 (33) Ireland	The Adaptive Physical Activity Study in Stroke (TAPAS): a feasibility sequential multiple assignment randomized trial	Assess feasibility of an adaptive mHealth physical-activity intervention and explore first-stage and subsequent adaptive treatment strategies.	Multicenter assessor-blinded feasibility SMART; community-based remote delivery.	Community-dwelling adults; mean time since stroke approximately 31 months. Ischemic and hemorrhagic stroke represented; some subtype responses unsure/missing.	50 (EX 28/LPA 22 at stage 1); 12 weeks
B. Intervention, comparator, eligible outcomes, and synthesis role.					
Study	Intervention, remote component, and dose	Comparator	Eligible review outcomes	Synthesis role/interpretation	
Fusari 2025 (28)	OnTrack wearable arm-activity feedback plus self-management coaching and usual care; daily use over 12 weeks. Promotes functional everyday arm use rather than an additional fixed exercise prescription.	Site-specific NHS multidisciplinary rehabilitation, including inpatient, community, or early supported discharge services. Content and intensity were not standardized; same usual-care pathway available to both arms.	QoL: EQ-5D-5L index and VAS. Contextual: MAL-14, SSEQ, mRS, adherence and feasibility.	Feasibility and descriptive evidence; clinical outcomes mainly medians/IQRs. Estimates the addition of OnTrack and coaching to routine rehabilitation, not exercise versus no care.	
Grau-Pellicer 2020 (29)	Multimodal PA/walking/exercise with FitLab, pedometer and bidirectional WhatsApp feedback; supervised sessions approximately 60 minutes twice weekly for 8 weeks; progression toward approximately 150 minutes/week where feasible.	Daily conventional rehabilitation over 3 months; active conventional comparator.	QoL: EQ-5D-5L descriptive problem score (lower is better). Contextual: BI, walking, mobility, activity/sedentary outcomes.	Small unblinded high-risk pilot. Selected mean-SD outcomes shown descriptively; nonstandard EQ-5D problem score not equated with a utility index.	
Linder 2015 (30)	Hand Mentor Pro robot-assisted upper-limb practice plus HEP, remote data upload and weekly telephone monitoring. Both arms prescribed 3 hours/day for 8 weeks.	Dose-matched upper-limb HEP with weekly telephone monitoring; active remote-supervised comparator.	Depression: CES-D. QoL-related: SIS domains. Contextual: functional SIS domains and adherence.	Active-vs-active incremental modality comparison. Approximately 27% of completers in each arm had CES-D >16. Adjusted within-group changes and time-by-treatment tests retained; no reconstructed between-group CI.	
Sun 2025 (31)	12-week individualized multidisciplinary telerehabilitation: PT/OT/speech and functional practice, videos, games, self-assessment, weekly video consultation, mental-health education and online psychological counseling.	Standard post-discharge care: rehabilitation manual and outpatient follow-up every 2 weeks.	Depression: HAMD. QoL: WHOQOL-BREF domains. Contextual: BI, mRS and safety.	Only directly analyzable depression contrast against standard care. Psychological counseling is a co-intervention; its exclusion leaves no such depression estimate. HAMD/BI mean-SD data and reported QoL coefficients kept distinct.	
van den Berg 2016 (32)	Caregiver-mediated walking/functional exercise with an iPad exercise platform, secure videoconferencing, Fitbit support, and weekly evaluation over 8 weeks.	Conventional multidisciplinary rehabilitation; active conventional comparator.	QoL-related: SIS domains. Patient HADS collected, but no separable patient anxiety estimate available in the main report. Contextual: NEADL, mobility, length of stay and caregiver results.	Proof-of-concept evidence. ITT primary mobility result did not establish superiority; per-protocol results exploratory. Caregiver HADS results are not patient depression/anxiety effects.	
Whiston 2026 (33)	Adaptive mHealth PA intervention: structured online exercise (EX) versus lifestyle physical activity (LPA), both supported by Fitbit feedback and remote coaching; adaptive tailoring over 12 weeks.	First-stage comparison of two active remote PA strategies, EX versus LPA; not exercise versus no intervention.	Combined distress: HADS total, not separate anxiety. QoL: SS-QOL and EQ-5D VAS. Contextual: steps, RNLI, FSS, adherence and safety.	Feasibility SMART. Group-specific adjusted marginal means and source-reported first-stage inference retained narratively. No reconstructed difference CI and no pooled psychiatric effect.	

Objectives are concise descriptions of the source-study aims, not new eligibility criteria. N denotes randomized participants and may differ from outcome-specific analysis denominators. Report titles identify the original studies; publications were not excluded because they were more than 10 years old.

Panel A and Panel B together constitute the study-level extraction table. Eligible outcomes and synthesis roles are deliberately separated. Functional outcomes are contextual; caregiver results are not patient psychiatric outcomes. Dose information is reported only where available. Comparator labels describe the actual care pathway: usual care can contain active rehabilitation. Abbreviations are defined in **Table 4**.

### Intervention characteristics

Interventions varied substantially in therapeutic content and implementation format. Remote components included wearable or digital feedback, mHealth applications, WhatsApp or app-based monitoring, telephone contact, video consultation, secure videoconferencing, therapist coaching, caregiver support, and an iPad-based exercise platform. Active content ranged from upper-limb practice and structured online exercise to walking, lifestyle physical activity, caregiver-mediated functional exercises, and multidisciplinary post-discharge rehabilitation. Sun et al. additionally provided mental-health education and online psychological counseling, while several other programs included self-management or behavioral coaching. Comparators ranged from standard care to dose-matched home exercise, conventional multidisciplinary rehabilitation, or another active remote physical-activity strategy (**Table 2**). Accordingly, the interventions were analyzed as complex remote packages rather than as isolated exercise prescriptions.

In Fusari et al., usual care consisted of site-specific NHS multidisciplinary stroke rehabilitation in inpatient, community, or early supported discharge services; its content and intensity were not standardized (28). OnTrack was added to that care, so this comparison does not represent activity versus no rehabilitation.

### Primary outcomes and main findings

Depression and psychological distress. Three studies contributed relevant information (30, 31, 33). Sun et al. reported lower post-intervention HAMD scores after 12 weeks than standard care; the aligned Hedges g was 0.93 (95% CI 0.64 to 1.22), a conventionally large standardized effect (31). This was the only directly analyzable depression contrast against standard care, and the intervention also contained mental-health education and online psychological counseling. Excluding Sun et al. therefore removed the entire direct standard-care depression evidence set; no alternative directly analyzable depression effect remained. In the active-vs-active Linder trial, adjusted within-group CES-D changes were -3.0 (95% CI -5.4 to -0.5) for home exercise and -4.0 (95% CI -6.3 to -1.7) for robot-assisted telerehabilitation plus home exercise; the time-by-treatment interaction was not significant (p=0.553). Because a covariance-adjusted between-group contrast and standard error were not reported, no between-group confidence interval was reconstructed (30). Whiston et al. reported group-specific adjusted marginal means for HADS total: 10.56 (95% CI 7.83 to 13.29) for EX and 14.00 (95% CI 11.13 to 16.87) for LPA post-intervention, and 9.88 (95% CI 7.08 to 12.69) and 12.33 (95% CI 8.90 to 15.77), respectively, at 3-month follow-up. The source reported no significant first-stage EX-versus-LPA differences or trends across secondary outcomes. Because covariance-adjusted between-group contrasts were not reported, between-group confidence intervals were not reconstructed (33). These source-reported findings were retained narratively and were not pooled (**Supplementary Table S2**).

Anxiety. No included study provided a separable patient anxiety effect suitable for synthesis. van den Berg et al. administered HADS to patients and caregivers, but the accessible main report did not provide a separable patient anxiety estimate; the 2.9-point per-protocol contrast reported under caregiver burden was not treated as a patient effect (32). Whiston et al. reported patient HADS total rather than an anxiety-subscale effect (33). This is an absence of separable outcome data, not indirect evidence of an anxiety treatment effect and not evidence that the interventions have no effect on anxiety.

Quality of life and health-related quality of life. All six studies reported a global measure or relevant quality-of-life domain (28–33). In usual- or standard-care comparisons, Fusari et al. reported feasibility medians for EQ-5D-5L without a clear favorable signal, and Sun et al. reported favorable adjusted WHOQOL-BREF coefficients across physical, mental, social, and environmental domains (beta 0.29 to 0.45) within a package that included psychological counseling (28, 31). Against active conventional rehabilitation, Grau-Pellicer et al. yielded an aligned EQ-5D-5L problem-score g of 1.26 (95% CI 0.50 to 2.02), conventionally large but derived from a small unblinded high-risk pilot, whereas van den Berg et al. reported mixed SIS domain findings (29, 32). In active remote comparisons, Linder et al. reported non-significant time-by-treatment interactions across SIS domains (p=0.197 to 0.992) (30). Whiston et al. reported group-specific adjusted marginal means for SS-QOL and EQ-5D VAS; the source reported no significant first-stage EX-versus-LPA differences or trends, and no between-group confidence intervals were reconstructed (33). These data were not pooled because instruments, constructs, time points, comparator structures, and effect metrics differed; sign alignment alone did not resolve those differences (**Supplementary Table S2**). Study-specific standardized depression and quality-of-life effects with analyzable mean-SD data are displayed in **Figure 2**.

**Figure 2:**
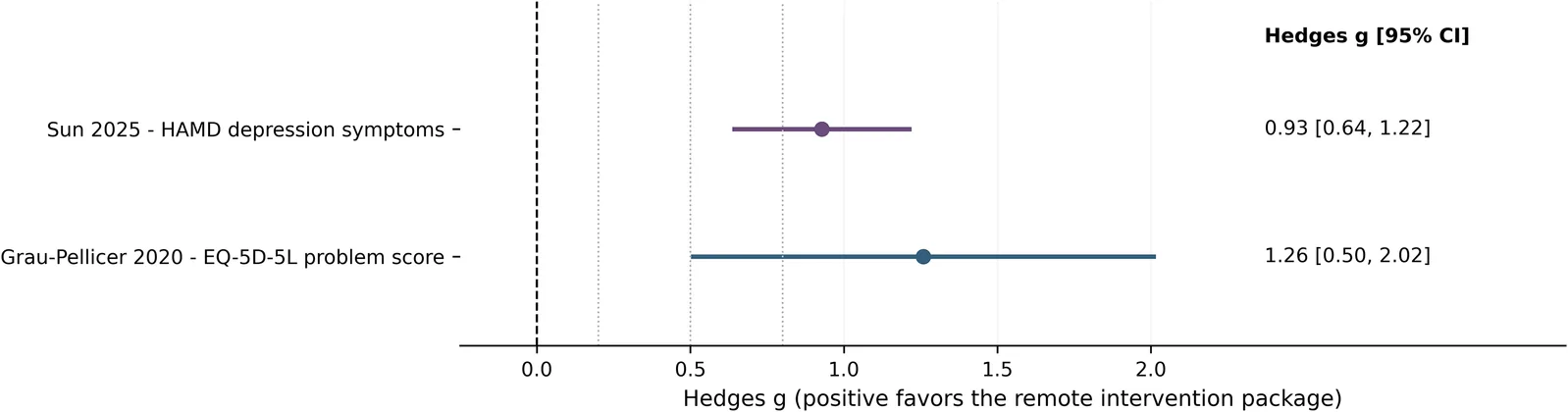
Exploratory standardized effects for depression and quality of life. HAMD (Sun et al., 12 weeks) and the EQ-5D-5L problem score (Grau-Pellicer et al., 3 months) are shown as separate study-specific contrasts, not as commensurable outcomes. Positive values favor the remote intervention package; no pooled estimate is shown. Conventional reference values of 0.2, 0.5, and 0.8 are statistical descriptors, not thresholds for clinical importance. The comparison in Sun et al. includes psychological counseling and the Grau-Pellicer trial uses an active conventional-rehabilitation comparator. Detailed functional/activity effects are presented separately in **Supplementary Figure S1**. CI, confidence interval; HAMD, Hamilton Depression Rating Scale; EQ-5D-5L, five-level EuroQol five-dimension questionnaire.

### Contextual rehabilitation outcomes

Functional recovery, activity, adherence, and safety findings were retained as exploratory rehabilitation context, not evidence of psychiatric efficacy. Detailed numerical results are presented in **Supplementary Results S1**, **Supplementary Table S2**, and **Supplementary Figure S1**. The studies did not test whether functional changes mediated depression, anxiety, or quality-of-life outcomes.

### Risk of bias, certainty, and evidence map

For the one representative review-relevant result assessed per trial with RoB 2, Grau-Pellicer et al. was judged at high overall risk and the other five trials had some concerns (**Table 3**; **Figure 3**). These judgments were not assumed to apply to every outcome in the corresponding trial. Concerns arose mainly from subjective outcome measurement in unblinded settings, missing outcome data, limited availability of prespecified analysis plans, and selective or exploratory reporting; feasibility design and active-comparator choice were considered separately as applicability and precision issues (**Supplementary Table S3**). GRADE certainty was very low for the evaluated depression/distress, quality-of-life, and contextual rehabilitation evidence bodies; anxiety was not rated because no separable patient anxiety effect was available. Downgrading reflected risk of bias, clinically heterogeneous interventions and comparators, limitations in applicability, small samples, and imprecision (**Supplementary Table S6**). The evidence map uses explicit text labels for Direct, Combined, Collected; no separable patient estimate, Reported, and Not reported status, with a machine-readable equivalent in **Supplementary Table S8** (**Figure 4**).

**Table 3:** Risk-of-bias summary.

Study	Overall RoB 2	Key concerns	Synthesis handling
Fusari et al., 2025 (28)	Some concerns	Representative EQ-5D-5L outcome was exploratory and self-reported; missing data and unavailable prespecified outcome analysis created some concerns.	Evidence map and descriptive/sensitivity synthesis.
Grau-Pellicer et al., 2020 (29)	High risk	Small unblinded pilot with subjective EQ-5D-5L measurement and concerns about deviations, missing data, and selective reporting.	Exploratory standardized display only; interpreted cautiously.
Linder et al., 2015 (30)	Some concerns	CES-D outcome in an active-vs-active trial; allocation and selective-reporting information were incomplete, although missing outcome data were limited.	Source-reported within-group changes and time-by-treatment interaction retained; no reconstructed between-group confidence interval.
Sun et al., 2025 (31)	Some concerns	Randomization, concealment, blinded assessment, and ITT were reported; the analysis plan was unavailable, creating some concern for selective reporting. Psychological support affects attribution, not the RoB 2 judgment.	Sole direct depression effect; very-low-certainty package-level evidence and co-intervention sensitivity.
van den Berg et al., 2016 (32)	Some concerns	Representative patient-reported SIS result in a proof-of-concept trial; missing outcome data and incomplete availability of prespecified analysis information created some concerns. The caregiver HADS result was not used as patient psychiatric evidence.	The patient SIS result was retained as QoL-related evidence and interpreted cautiously; caregiver HADS findings were not treated as patient depression or anxiety effects.
Whiston et al., 2026 (33)	Some concerns	Feasibility SMART with missing data, subjective HADS measurement, and model-based exploratory outcomes.	Group-specific adjusted marginal means and source-reported first-stage inference retained narratively; no reconstructed difference confidence interval.

Overall judgments are based on one representative review-relevant result assessed for each trial with the Cochrane RoB 2 framework; these judgments are not assumed to apply to every outcome in the trial. Detailed domain judgments are provided in **Supplementary Table S3**, and synthesis-level certainty is reported separately in **Supplementary Table S6**. RoB 2, Risk of Bias 2; SMART, sequential multiple assignment randomized trial.

**Figure 3:**
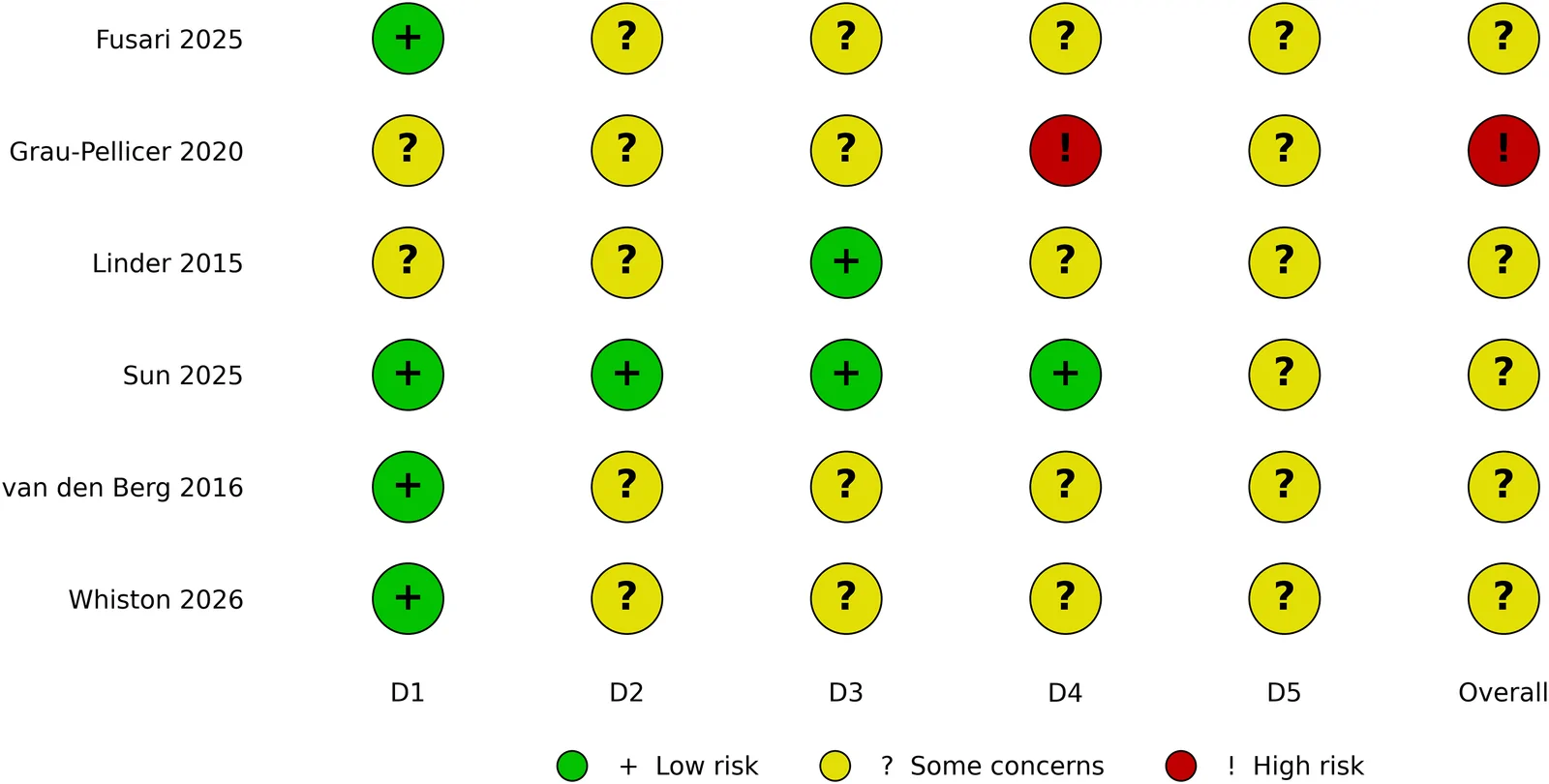
Risk-of-bias traffic-light plot for one representative review-relevant result per trial using the Cochrane RoB 2 framework. The judgments are not assumed to apply to all outcomes reported by the same trial. Green, yellow, and red circles are accompanied by the symbols +,?, and! and category labels for low risk, some concerns, and high risk, respectively. D1, randomization process; D2, deviations from intended interventions; D3, missing outcome data; D4, outcome measurement; D5, selection of the reported result.

**Figure 4:**
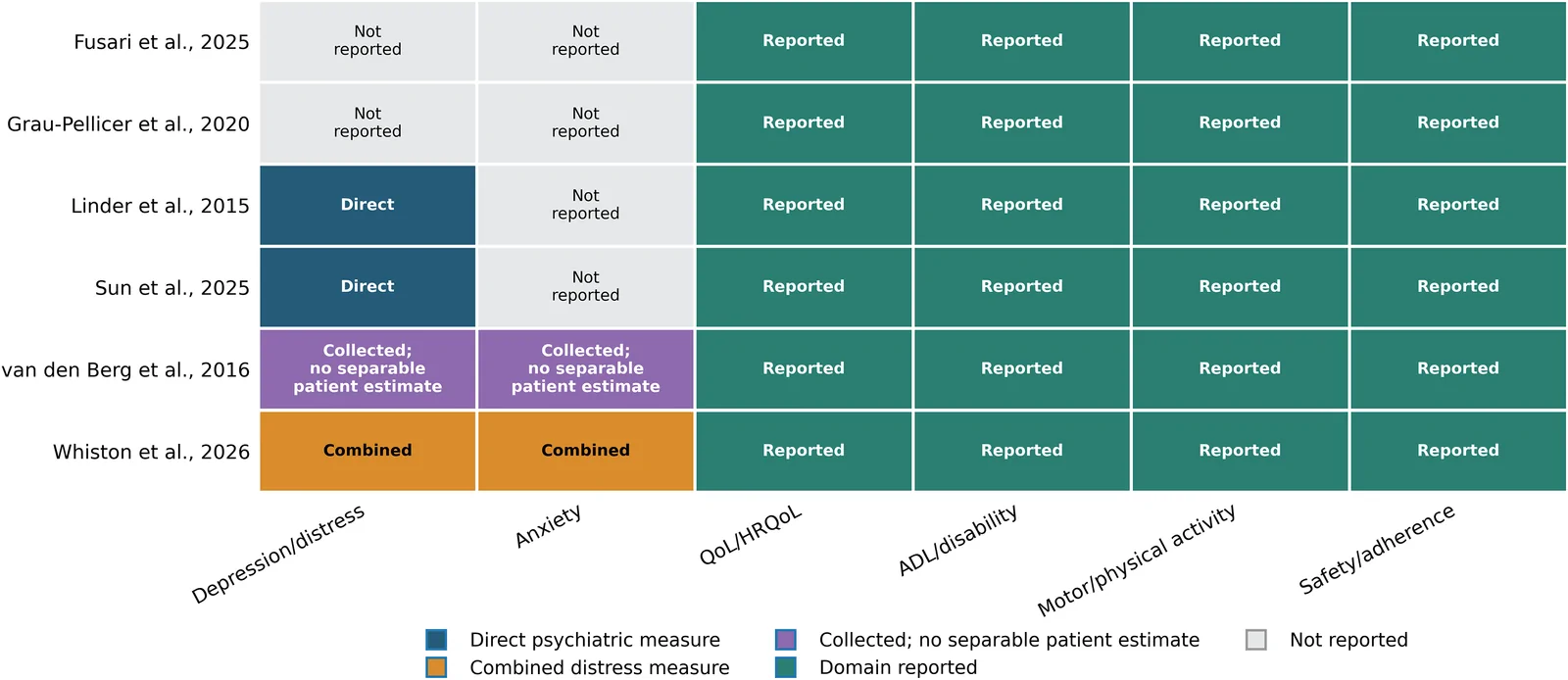
Accessible evidence map of outcome domains in the six included studies. Every cell contains an explicit status label: Direct indicates a standalone depression or anxiety measure; Combined indicates a non-separable patient distress measure; Collected; no separable patient estimate indicates that the construct was measured but no separable patient estimate was available; Reported indicates that the non-psychiatric domain was reported; and Not reported indicates absence of eligible data. The map does not indicate effect direction, magnitude, or certainty. A machine-readable equivalent is provided in **Supplementary Table S8**. QoL/HRQoL, quality of life or health-related quality of life; ADL, activities of daily living. Functional and activity columns provide rehabilitation context and are not proxies for psychiatric benefit.

## Discussion

### Principal findings

This review identified a small and heterogeneous randomized evidence base for remotely supervised exercise- and physical activity-based rehabilitation packages targeting mental-health-relevant outcomes after stroke. The apparent depression benefit was supported entirely by Sun et al., the only directly analyzable standard-care comparison, and that program included online psychological counseling (31). Although the HAMD effect was conventionally large, its certainty was very low and exclusion of the trial left no direct standard-care depression effect. The depression signal is therefore fragile and cannot be attributed to exercise alone. Quality-of-life findings were mixed across global and domain-specific measures, no separable patient anxiety effect was available, and no pooled primary estimate was defensible.

These findings should be interpreted alongside broader stroke rehabilitation evidence. Telerehabilitation can deliver rehabilitation services and may produce selected functional outcomes comparable with in-clinic therapy (9, 10), while physical fitness training after stroke has a clearer evidence base for mobility and fitness than for psychiatric endpoints (11, 17). Remote delivery has a plausible logistical rationale, but the included trials did not measure reach, equitable access, travel burden, or service penetration. We therefore do not infer that the reviewed interventions expanded access; this remains an implementation hypothesis for future study.

### Interpretation for psychiatry and rehabilitation

Several mechanisms could link activity with emotional recovery, but they should be distinguished from explanations demonstrated by these trials. Exercise-related neuroplasticity, including neurotrophic signaling, and regulation of inflammatory and hypothalamic-pituitary-adrenal stress responses are proposed biological pathways in the broader depression literature (12). Their relevance to these particular remote rehabilitation packages remains untested. Behavioral pathways may be more proximal: achievable activity goals and feedback could restore confidence after loss of function, while successful practice could reduce avoidance and strengthen self-efficacy (12, 13). For anxiety, safely experiencing exertional arousal may lessen fear of bodily sensations, but that hypothesis cannot be tested using a combined HADS score alone (13). Greater independence and participation could improve quality of life without necessarily reducing depression or anxiety. Repeated therapist contact, caregiver assistance, and psychological counseling could also influence well-being independently of exercise. Accordingly, neither a functional gain nor a favorable package-level outcome identifies an exercise-specific psychiatric mechanism (15, 16).

None of the included trials performed a mediation analysis linking these pathways to psychiatric change. Such testing would require repeated measurements of candidate mediators before the psychiatric endpoint and designs that separate exercise dose from contact, coaching, and psychological support. The present results therefore support hypotheses for future trials, not causal claims about neurobiological or psychosocial mediation.

These comparator categories address different questions. A package-versus-standard-care contrast includes differences in therapeutic contact and co-interventions, whereas active remote comparisons estimate incremental differences between rehabilitation strategies. A non-significant comparison does not establish equivalence. HADS total is not interchangeable with a depression or anxiety subscale, and an individual SIS domain is not a global quality-of-life score. Differences in stroke stage, follow-up, exercise content, and outcome reporting further limit cross-trial interpretation. Multiple favorable outcomes from one small trial are not independent confirmations of efficacy; selective emphasis on such findings would overstate this exploratory evidence (21, 22).

Feasibility trials, proof-of-concept studies, and adaptive SMART designs are appropriate during early intervention development. The ORBIT and NIH Stage models explicitly support iterative optimization, feasibility testing, and mechanism-focused work before definitive efficacy and implementation trials (35, 36). Our concern is therefore not that these designs were inappropriate, but that their developmental purpose, active comparators, small samples, and model-based estimates limit the confirmatory claims that can be drawn from them. The evidence map and study-specific contrasts preserve their early signals without converting them into an overprecise pooled effect.

### Clinical implications

For clinicians and service planners, the current evidence supports remotely supervised exercise or physical activity as a potential component of multidisciplinary stroke rehabilitation, not as a stand-alone evidence-based treatment for post-stroke depression or anxiety. Outside stroke, a systematic review of exercise therapy in people with multimorbidity reported improvements in depressive symptoms and health-related quality of life, with low-certainty evidence (37). That broader evidence does not establish the efficacy of remotely supervised exercise for post-stroke psychiatric disorders. Stroke survivors with clinically significant depression or anxiety should continue to receive evidence-based psychiatric, psychological, and, where indicated, pharmacological care. Any mental-health benefit of a remote rehabilitation package should be interpreted alongside its psychological and behavioral components.

### Strengths and limitations

Strengths of this review include PROSPERO registration, a PRISMA-based search across six databases, independent dual screening and extraction, RoB 2 assessment, GRADE certainty assessment, transparent final eligibility adjudication, and provision of canonical source-traceable datasets and reproducible figure code. The review was deliberately scoped to align with mental-health and psychiatric rehabilitation outcomes rather than physical-function outcomes alone.

Several limitations should be emphasized. Six trials were included, and the direct depression signal within this set depended on one multicomponent trial with psychological counseling. Most trials were not powered for psychiatric outcomes, and none recruited participants on the basis of a confirmed depressive or anxiety disorder; low symptom burden in some samples may have reduced the scope for improvement, whereas the Sun sample had elevated mean baseline HAMD scores without diagnostic enrichment. Instruments, time points, comparators, and effect metrics varied substantially. Some studies reported medians, adjusted within-group changes, interaction tests, or SMART marginal means rather than complete group means and standard deviations; unsupported between-group confidence intervals were not reconstructed. Conventional small, moderate, and large labels do not establish clinical importance. The review could not isolate exercise from psychological, coaching, caregiver, or implementation components, and the trials did not report access or reach metrics. The executed database strategies required a depression/anxiety/quality-of-life outcome concept, and the original workflow did not document trial-registry searching or formal backward/forward citation tracking; trials in which these outcomes were secondary or absent from titles or abstracts may therefore have been missed. Although the original database strategies required a depression/anxiety/quality-of-life concept, a supplementary higher-sensitivity verification search without mandatory outcome terms identified no additional eligible study. The possibility of incomplete retrieval cannot be eliminated entirely in any systematic review, but the supplementary search directly addressed the principal sensitivity concern associated with the original outcome restriction. Fewer than 10 comparable studies were available, so funnel plots and formal small-study-effect tests were inappropriate. Not pooling reduced statistical compactness but avoided a misleading summary estimate.

### Implications for future research

Future trials should recruit or stratify participants according to baseline depression and anxiety, prespecify psychiatric and health-related quality-of-life outcomes, and report complete means, standard deviations, change scores, and clinically interpretable responder thresholds. Validated instruments such as PHQ-9, HADS subscales, HAMD, GAD-7, EQ-5D, WHOQOL-BREF, or SS-QOL should be used consistently. Factorial, component, or sequential optimization designs could separate the contributions of exercise dose, remote supervision, psychological support, education, and coaching before confirmatory comparative-effectiveness trials. Reports should detail exercise dose and progression, supervision frequency, feedback modality, technology support, co-interventions, adherence, adverse events, acceptability, reach, and equity in accordance with reproducible intervention-reporting guidance (16). Abbreviations used in the manuscript and tables are listed in **Table 4**.

**Table 4:** Abbreviations used in the manuscript and tables.

Abbreviation	Meaning	Abbreviation	Meaning
ADL	Activities of daily living	NHS	National Health Service
BI	Barthel Index	NIH	National Institutes of Health
CES-D	Center for Epidemiologic Studies Depression Scale	ORBIT	Obesity-Related Behavioral Intervention Trials
CI	Confidence interval	OT	Occupational therapy
EQ-5D-5L	Five-level EuroQol five-dimension questionnaire	PA	Physical activity
EX	Structured online exercise	PHQ-9	Patient Health Questionnaire-9
FAC	Functional Ambulation Categories	PP	Per-protocol
FSS	Fatigue Severity Scale	PRISMA	Preferred Reporting Items for Systematic Reviews and Meta-Analyses
GAD-7	Generalized Anxiety Disorder 7-item scale	PROSPERO	International Prospective Register of Systematic Reviews
GRADE	Grading of Recommendations Assessment, Development and Evaluation	PT	Physical therapy
HADS	Hospital Anxiety and Depression Scale	QoL	Quality of life
HAMD	Hamilton Depression Rating Scale	RCT	Randomized controlled trial
HEP	Home exercise program	RNLI	Reintegration to Normal Living Index
HRQoL	Health-related quality of life	RoB 2	Revised Cochrane risk-of-bias tool for randomized trials
I/C	Intervention/control	RR	Risk ratio
IADL	Instrumental activities of daily living	SD	Standard deviation
IQR	Interquartile range	SIS	Stroke Impact Scale
ITT	Intention-to-treat	SMART	Sequential multiple assignment randomized trial
LPA	Lifestyle physical activity	SMD	Standardized mean difference
MAL-14	14-item Motor Activity Log	SS-QOL	Stroke-Specific Quality of Life Scale
MD	Mean difference	SSEQ	Stroke Self-Efficacy Questionnaire
mHealth	Mobile health	SWiM	Synthesis Without Meta-analysis
MMSE	Mini-Mental State Examination	TAPAS	The Adaptive Physical Activity Study in Stroke
mRS	Modified Rankin Scale	VAS	Visual analogue scale
NEADL	Nottingham Extended Activities of Daily Living	WHOQOL-BREF	World Health Organization Quality of Life Brief Version

## Conclusion

The included randomized evidence for depression/distress and quality of life after remotely supervised exercise- or physical activity-based stroke rehabilitation remains sparse, heterogeneous, and very low certainty. No separable patient anxiety effect was available. The only direct depression signal against standard care came from one multicomponent trial that included psychological counseling, and excluding that trial left no direct analyzable depression effect. Current findings therefore support further evaluation of complex remote rehabilitation packages, not a conclusion that exercise alone treats post-stroke depression or anxiety.

## Data Availability

The original contributions presented in the study are included in the article/**Supplementary Material**. Further inquiries can be directed to the corresponding author.
