# Supplementary material for: Remote supervised exercise- and physical activity-based rehabilitation for post-stroke depression, anxiety, and quality of life: a systematic review, evidence map, and exploratory quantitative synthesis
Source: Front Psychiatry. 2026 Oct 16;17:1888180. doi: 10.3389/fpsyt.2026.1888180 (PMC13719302; doi:10.3389/fpsyt.2026.1888180)
Supplement: Supplementary file 1 [file SupplementaryFile1.docx]

# Supplementary Material

Remote supervised exercise- and physical activity-based rehabilitation for post-stroke depression, anxiety, and quality of life

# Table S1. Recorded database search strategies and original record counts.

| **Database** | **Platform** | **Records identified** | **Search strategy** |
| --- | --- | --- | --- |
| PubMed | MEDLINE via PubMed | 454 | ((stroke[MeSH Terms] OR stroke*[tiab] OR cerebrovascular accident*[tiab] OR poststroke[tiab] OR post-stroke[tiab]) AND (telerehabilitation[tiab] OR tele-rehabilitation[tiab] OR remote rehabilitation[tiab] OR home-based rehabilitation[tiab] OR mHealth[tiab] OR mobile health[tiab] OR app[tiab] OR web-based[tiab] OR video[tiab] OR telephone[tiab] OR wearable*[tiab] OR robot*[tiab]) AND (exercise[MeSH Terms] OR exercise therap*[tiab] OR physical activit*[tiab] OR rehabilitation[tiab] OR training[tiab] OR motor training[tiab]) AND (depress*[tiab] OR anxiety[tiab] OR quality of life[tiab] OR HRQoL[tiab] OR EQ-5D[tiab] OR HADS[tiab] OR HAMD[tiab] OR CES-D[tiab] OR Stroke Impact Scale[tiab]) AND (randomized controlled trial[pt] OR randomized[tiab] OR randomised[tiab] OR randomly[tiab] OR trial[tiab])) |
| Web of Science | Web of Science Core Collection | 691 | TS=((stroke* OR "cerebrovascular accident*" OR poststroke OR "post-stroke") AND (telerehabilitation OR "tele-rehabilitation" OR "remote rehabilitation" OR "home-based rehabilitation" OR mHealth OR "mobile health" OR app OR "web-based" OR video OR telephone OR wearable* OR robot*) AND (exercise OR "exercise therapy" OR "physical activit*" OR rehabilitation OR training OR "motor training") AND (depress* OR anxiety OR "quality of life" OR HRQoL OR "EQ-5D" OR HADS OR HAMD OR "CES-D" OR "Stroke Impact Scale") AND (random* OR trial)) |
| SPORTDiscus | EBSCOhost SPORTDiscus | 105 | ((stroke* OR "cerebrovascular accident*" OR poststroke OR "post-stroke") AND (telerehabilitation OR "remote rehabilitation" OR "home-based rehabilitation" OR mHealth OR app OR wearable* OR robot* OR video OR telephone) AND (exercise OR "physical activity" OR rehabilitation OR training) AND (depress* OR anxiety OR "quality of life" OR HRQoL OR HADS OR HAMD OR "CES-D") AND (random* OR trial)) |
| Cochrane Library | CENTRAL and Cochrane Library | 242 | ([stroke OR post-stroke OR poststroke OR cerebrovascular accident] AND [telerehabilitation OR remote rehabilitation OR home-based rehabilitation OR mHealth OR app OR web OR video OR telephone OR wearable OR robot] AND [exercise OR physical activity OR rehabilitation OR training] AND [depression OR anxiety OR quality of life OR HRQoL OR HADS OR HAMD OR CES-D]) in Trials |
| Embase | Embase | 644 | ('stroke'/exp OR stroke*:ti,ab OR 'cerebrovascular accident*':ti,ab OR poststroke:ti,ab OR 'post-stroke':ti,ab) AND (telerehabilitation:ti,ab OR 'tele-rehabilitation':ti,ab OR 'remote rehabilitation':ti,ab OR 'home-based rehabilitation':ti,ab OR mhealth:ti,ab OR app:ti,ab OR 'web-based':ti,ab OR video:ti,ab OR telephone:ti,ab OR wearable*:ti,ab OR robot*:ti,ab) AND ('exercise'/exp OR 'exercise therapy':ti,ab OR 'physical activity':ti,ab OR rehabilitation:ti,ab OR training:ti,ab) AND (depress*:ti,ab OR anxiety:ti,ab OR 'quality of life':ti,ab OR hrqol:ti,ab OR 'eq-5d':ti,ab OR hads:ti,ab OR hamd:ti,ab OR 'ces-d':ti,ab) AND ('randomized controlled trial'/exp OR random*:ti,ab OR trial:ti,ab) |
| Scopus | Scopus | 321 | TITLE-ABS-KEY((stroke* OR "cerebrovascular accident*" OR poststroke OR "post-stroke") AND (telerehabilitation OR "tele-rehabilitation" OR "remote rehabilitation" OR "home-based rehabilitation" OR mHealth OR "mobile health" OR app OR "web-based" OR video OR telephone OR wearable* OR robot*) AND (exercise OR "exercise therapy" OR "physical activit*" OR rehabilitation OR training OR "motor training") AND (depress* OR anxiety OR "quality of life" OR HRQoL OR "EQ-5D" OR HADS OR HAMD OR "CES-D" OR "Stroke Impact Scale") AND (random* OR trial)) |

Searches were conducted from database inception to 1 March 2026 in PubMed, Web of Science, SPORTDiscus, Cochrane Library, Embase, and Scopus. Platforms and record counts are reported before de-duplication. The strategies are reproduced as recorded for the original outcome-filtered search; the reported counts do not represent a new search without outcome terms. The original search workflow did not document trial-registry or formal backward/forward citation searching. The outcome block restricts retrieval to the review focus but may miss secondary or poorly indexed outcomes. No higher-sensitivity search is claimed for these counts. The outcome restriction and the absence of documented registry/citation searching limit sensitivity. Cochrane wording is retained in the form recorded in the original search documentation; it is not represented as a newly validated Search Manager history.

# Table S2. Source-traceable outcome-level extraction for the six included studies.

| **Study** | **Source location** | **Outcome domain** | **Outcome** | **Result type** | **Result** | **95% CI** | **Interpretation** | **Synthesis note** |
| --- | --- | --- | --- | --- | --- | --- | --- | --- |
| Fusari et al., 2025 | Article Table 5 | Motor function/physical activity | MAL-14 how much, 12 weeks | Median difference I-C | 0.25 | NR | Direction follows the source scale; retained narratively. | Sensitivity/narrative: feasibility trial and median/IQR data |
| Fusari et al., 2025 | Article Table 5 | Motor function/physical activity | MAL-14 how well, 12 weeks | Median difference I-C | 0.143 | NR | Direction follows the source scale; retained narratively. | Sensitivity/narrative: feasibility trial and median/IQR data |
| Fusari et al., 2025 | Article Table 5 | Other | Modified Rankin Scale, 12 weeks | Median difference I-C | 0 | NR | Direction follows the source scale; retained narratively. | Sensitivity/narrative: feasibility trial and median/IQR data |
| Fusari et al., 2025 | Article Table 5 | Other | Stroke Self-Efficacy Questionnaire (SSEQ), 12 weeks | Median difference I-C | -1 | NR | Direction follows the source scale; retained narratively. | Sensitivity/narrative: feasibility trial and median/IQR data |
| Fusari et al., 2025 | Article Table 5 | Quality of life/HRQoL or SIS domain | EQ-5D-5L VAS, 12 weeks | Median difference I-C | -18 | NR | Direction follows the source scale; retained narratively. | Sensitivity/narrative: feasibility trial and median/IQR data |
| Fusari et al., 2025 | Article Table 5 | Quality of life/HRQoL or SIS domain | EQ-5D-5L index, 12 weeks | Median difference I-C | -0.014 | NR | Direction follows the source scale; retained narratively. | Sensitivity/narrative: feasibility trial and median/IQR data |
| Grau-Pellicer et al., 2020 | Article Table 2 | ADL/disability/participation | Barthel Index, 3 months | Aligned post-intervention mean difference | 11.09 | 2.3627 to 19.8173 | Positive values favor the remote intervention package where an aligned effect was derived. | Narrative/sensitivity |
| Grau-Pellicer et al., 2020 | Article Table 2 | Motor function/physical activity | 10MWT comfortable speed, m/s, 3 months | Aligned post-intervention mean difference | 0.34 | 0.1284 to 0.5516 | Positive values favor the remote intervention package where an aligned effect was derived. | Narrative/sensitivity |
| Grau-Pellicer et al., 2020 | Article Table 2 | Motor function/physical activity | 10MWT fast speed, m/s, 3 months | Aligned post-intervention mean difference | 0.48 | 0.2001 to 0.7599 | Positive values favor the remote intervention package where an aligned effect was derived. | Narrative/sensitivity |
| Grau-Pellicer et al., 2020 | Article Table 2 | Motor function/physical activity | 6-minute walk test, m, 3 months | Aligned post-intervention mean difference | 109.48 | 35.3652 to 183.59 | Positive values favor the remote intervention package where an aligned effect was derived. | Narrative/sensitivity |
| Grau-Pellicer et al., 2020 | Article Table 2 | Motor function/physical activity | Community ambulation, min/day, 3 months | Aligned post-intervention mean difference | 41.88 | 7.2439 to 76.5161 | Positive values favor the remote intervention package where an aligned effect was derived. | Narrative/sensitivity |
| Grau-Pellicer et al., 2020 | Article Table 2 | Motor function/physical activity | Sitting time, hours/day, 3 months | Aligned post-intervention mean difference | 5.5 | 2.0162 to 8.9838 | Positive values favor the remote intervention package where an aligned effect was derived. | Narrative/sensitivity |
| Grau-Pellicer et al., 2020 | Article Table 2 | Motor function/physical activity | Timed Up and Go, seconds, 3 months | Aligned post-intervention mean difference | 12.49 | -0.3596 to 25.3396 | Positive values favor the remote intervention package where an aligned effect was derived. | Narrative/sensitivity |
| Grau-Pellicer et al., 2020 | Article Table 2 | Quality of life/HRQoL or SIS domain | EQ-5D-5L descriptive problem score, 3 months | Aligned post-intervention mean difference | 4.11 | 1.7579 to 6.4621 | Positive values favor the remote intervention package where an aligned effect was derived. | Sensitivity: mixed mHealth/conventional rehab and special lower-is-better EQ-5D problem score |
| Linder et al., 2015 | Article Table 2 | Depression/anxiety/psychological symptoms | CES-D change, end of treatment | Adjusted within-group change and time x group interaction | HEP: -3.0 (95% CI -5.4 to -0.5); Robot+HEP: -4.0 (95% CI -6.3 to -1.7); interaction p=0.553 | See result | Not applicable: source-reported within-group changes and an interaction test. | Active-vs-active comparison. The interaction p value is the source-reported between-group inference. A covariance-adjusted between-group contrast and standard error were not reported, so no between-group confidence interval was reconstructed. |
| Linder et al., 2015 | Article Table 2 | Quality of life/HRQoL or SIS domain | SIS ADL/IADL change, end of treatment | Adjusted within-group change and time x group interaction | HEP: 10.0 (95% CI 5.1 to 14.9); Robot+HEP: 10.0 (95% CI 5.2 to 14.8); interaction p=0.992 | See result | Not applicable: source-reported within-group changes and an interaction test. | Active-vs-active comparison. The interaction p value is the source-reported between-group inference. A covariance-adjusted between-group contrast and standard error were not reported, so no between-group confidence interval was reconstructed. |
| Linder et al., 2015 | Article Table 2 | Quality of life/HRQoL or SIS domain | SIS Communication change, end of treatment | Adjusted within-group change and time x group interaction | HEP: 7.8 (95% CI 3.5 to 12.0); Robot+HEP: 5.7 (95% CI 1.6 to 9.9); interaction p=0.498 | See result | Not applicable: source-reported within-group changes and an interaction test. | Active-vs-active comparison. The interaction p value is the source-reported between-group inference. A covariance-adjusted between-group contrast and standard error were not reported, so no between-group confidence interval was reconstructed. |
| Linder et al., 2015 | Article Table 2 | Quality of life/HRQoL or SIS domain | SIS Hand Function change, end of treatment | Adjusted within-group change and time x group interaction | HEP: 26.5 (95% CI 18.7 to 34.4); Robot+HEP: 22.7 (95% CI 14.9 to 30.5); interaction p=0.495 | See result | Not applicable: source-reported within-group changes and an interaction test. | Active-vs-active comparison. The interaction p value is the source-reported between-group inference. A covariance-adjusted between-group contrast and standard error were not reported, so no between-group confidence interval was reconstructed. |
| Linder et al., 2015 | Article Table 2 | Quality of life/HRQoL or SIS domain | SIS Meaningful Activities change, end of treatment | Adjusted within-group change and time x group interaction | HEP: 15.2 (95% CI 9.0 to 21.5); Robot+HEP: 14.9 (95% CI 8.7 to 21.0); interaction p=0.930 | See result | Not applicable: source-reported within-group changes and an interaction test. | Active-vs-active comparison. The interaction p value is the source-reported between-group inference. A covariance-adjusted between-group contrast and standard error were not reported, so no between-group confidence interval was reconstructed. |
| Linder et al., 2015 | Article Table 2 | Quality of life/HRQoL or SIS domain | SIS Memory change, end of treatment | Adjusted within-group change and time x group interaction | HEP: 6.3 (95% CI 0.8 to 11.7); Robot+HEP: 2.6 (95% CI -2.7 to 8.0); interaction p=0.346 | See result | Not applicable: source-reported within-group changes and an interaction test. | Active-vs-active comparison. The interaction p value is the source-reported between-group inference. A covariance-adjusted between-group contrast and standard error were not reported, so no between-group confidence interval was reconstructed. |
| Linder et al., 2015 | Article Table 2 | Quality of life/HRQoL or SIS domain | SIS Mobility change, end of treatment | Adjusted within-group change and time x group interaction | HEP: 6.4 (95% CI 1.8 to 11.0); Robot+HEP: 8.4 (95% CI 3.9 to 12.9); interaction p=0.541 | See result | Not applicable: source-reported within-group changes and an interaction test. | Active-vs-active comparison. The interaction p value is the source-reported between-group inference. A covariance-adjusted between-group contrast and standard error were not reported, so no between-group confidence interval was reconstructed. |
| Linder et al., 2015 | Article Table 2 | Quality of life/HRQoL or SIS domain | SIS Mood change, end of treatment | Adjusted within-group change and time x group interaction | HEP: 3.3 (95% CI -1.4 to 8.1); Robot+HEP: 7.3 (95% CI 2.7 to 11.9); interaction p=0.235 | See result | Not applicable: source-reported within-group changes and an interaction test. | Active-vs-active comparison. The interaction p value is the source-reported between-group inference. A covariance-adjusted between-group contrast and standard error were not reported, so no between-group confidence interval was reconstructed. |
| Linder et al., 2015 | Article Table 2 | Quality of life/HRQoL or SIS domain | SIS Strength change, end of treatment | Adjusted within-group change and time x group interaction | HEP: 12.4 (95% CI 7.0 to 17.8); Robot+HEP: 9.5 (95% CI 4.2 to 14.7); interaction p=0.442 | See result | Not applicable: source-reported within-group changes and an interaction test. | Active-vs-active comparison. The interaction p value is the source-reported between-group inference. A covariance-adjusted between-group contrast and standard error were not reported, so no between-group confidence interval was reconstructed. |
| Linder et al., 2015 | Article Table 2 | Quality of life/HRQoL or SIS domain | SIS Stroke Recovery change, end of treatment | Adjusted within-group change and time x group interaction | HEP: 6.0 (95% CI 1.2 to 10.8); Robot+HEP: 10.5 (95% CI 5.7 to 15.2); interaction p=0.197 | See result | Not applicable: source-reported within-group changes and an interaction test. | Active-vs-active comparison. The interaction p value is the source-reported between-group inference. A covariance-adjusted between-group contrast and standard error were not reported, so no between-group confidence interval was reconstructed. |
| Sun et al., 2025 | Abstract/results text | ADL/disability/participation | Barthel Index, 12 weeks | Aligned post-intervention mean difference | 13.8 | 10.6009 to 16.9991 | Positive values favor the remote intervention package where an aligned effect was derived. | Narrative/sensitivity |
| Sun et al., 2025 | Abstract/results text | ADL/disability/participation | mRS 0-2 favorable outcome, 12 weeks | Intervention-versus-control risk ratio | 1.2097 | 0.9997 to 1.4638 | RR >1 favors the remote package; RR=1 indicates no between-group difference. | Narrative/sensitivity; favorable outcome RR, intervention versus control |
| Sun et al., 2025 | Abstract/results text | Depression/anxiety/psychological symptoms | HAMD, 12 weeks | Aligned post-intervention mean difference | 3.6 | 2.529 to 4.671 | Positive values favor the remote intervention package where an aligned effect was derived. | Package-level evidence; this trial was excluded in the psychological-support sensitivity analysis. No pooled estimate was recalculated. |
| Sun et al., 2025 | Article Table 2 | Quality of life/HRQoL or SIS domain | WHOQOL-BREF environmental field, adjusted beta | Reported adjusted coefficient | 0.29 | 0.06 to 0.52 | Positive coefficient favors the remote intervention package. | Package-level evidence; this trial was excluded in the psychological-support sensitivity analysis. No pooled estimate was recalculated. |
| Sun et al., 2025 | Article Table 2 | Quality of life/HRQoL or SIS domain | WHOQOL-BREF mental function, adjusted beta | Reported adjusted coefficient | 0.45 | 0.23 to 0.67 | Positive coefficient favors the remote intervention package. | Package-level evidence; this trial was excluded in the psychological-support sensitivity analysis. No pooled estimate was recalculated. |
| Sun et al., 2025 | Article Table 2 | Quality of life/HRQoL or SIS domain | WHOQOL-BREF physical function, adjusted beta | Reported adjusted coefficient | 0.38 | 0.12 to 0.64 | Positive coefficient favors the remote intervention package. | Package-level evidence; this trial was excluded in the psychological-support sensitivity analysis. No pooled estimate was recalculated. |
| Sun et al., 2025 | Article Table 2 | Quality of life/HRQoL or SIS domain | WHOQOL-BREF social relation, adjusted beta | Reported adjusted coefficient | 0.32 | 0.07 to 0.57 | Positive coefficient favors the remote intervention package. | Package-level evidence; this trial was excluded in the psychological-support sensitivity analysis. No pooled estimate was recalculated. |
| Van den Berg et al., 2016 | Article abstract/results text | ADL/disability/participation | Nottingham Extended ADL, 12 weeks, per-protocol | Aligned adjusted mean difference | 3 | 0.3 to 5.8 | Positive values favor the remote intervention package where an aligned effect was derived. | Sensitivity/narrative: proof-of-concept trial; ITT preferred, PP exploratory |
| Van den Berg et al., 2016 | Article abstract/results text | ADL/disability/participation | Nottingham Extended ADL, 8 weeks, per-protocol | Aligned adjusted mean difference | 3.6 | 0.8 to 6.3 | Positive values favor the remote intervention package where an aligned effect was derived. | Sensitivity/narrative: proof-of-concept trial; ITT preferred, PP exploratory |
| Van den Berg et al., 2016 | Article results text | Caregiver psychosocial outcomes | Caregiver HADS anxiety/depression burden, 12 weeks, per-protocol | Aligned adjusted mean difference | 2.9 | -0.1 to 5.8 | Positive values favor the remote intervention package where an aligned effect was derived. | Caregiver-burden result in the source main text; not used as a patient depression or anxiety effect. |
| Van den Berg et al., 2016 | Article results text | Motor function/physical activity | Timed Up and Go, 12 weeks, ITT | Aligned adjusted mean difference | -8 | -15.3 to -0.8 | Positive values favor the remote intervention package where an aligned effect was derived. | Sensitivity/narrative: proof-of-concept trial; ITT preferred, PP exploratory |
| Van den Berg et al., 2016 | Article results text | Quality of life/HRQoL or SIS domain | SIS Communication, 12 weeks, per-protocol | Aligned adjusted mean difference | 7.3 | 1 to 13.6 | Positive values favor the remote intervention package where an aligned effect was derived. | Sensitivity/narrative: proof-of-concept trial; ITT preferred, PP exploratory |
| Van den Berg et al., 2016 | Article results text | Quality of life/HRQoL or SIS domain | SIS Communication, 8 weeks, per-protocol | Aligned adjusted mean difference | 7.7 | 1.3 to 14 | Positive values favor the remote intervention package where an aligned effect was derived. | Sensitivity/narrative: proof-of-concept trial; ITT preferred, PP exploratory |
| Van den Berg et al., 2016 | Article results text | Quality of life/HRQoL or SIS domain | SIS Memory, 12 weeks, ITT | Aligned adjusted mean difference | 11.2 | 4.3 to 18.2 | Positive values favor the remote intervention package where an aligned effect was derived. | Sensitivity/narrative: proof-of-concept trial; ITT preferred, PP exploratory |
| Van den Berg et al., 2016 | Article results text | Quality of life/HRQoL or SIS domain | SIS Memory, 12 weeks, per-protocol | Aligned adjusted mean difference | 15.4 | 7.3 to 23.5 | Positive values favor the remote intervention package where an aligned effect was derived. | Sensitivity/narrative: proof-of-concept trial; ITT preferred, PP exploratory |
| Van den Berg et al., 2016 | Article results text | Quality of life/HRQoL or SIS domain | SIS Mobility, 8 weeks, ITT | Not reported in accessible main report | Not reported in accessible main text | NA | Not applicable. | Sensitivity/narrative: proof-of-concept trial; ITT preferred, PP exploratory |
| Van den Berg et al., 2016 | Article abstract/results text | Quality of life/HRQoL or SIS domain | SIS Mobility, 8 weeks, per-protocol | Aligned adjusted mean difference | 9.8 | -0.4 to 20.1 | Positive values favor the remote intervention package where an aligned effect was derived. | Sensitivity/narrative: proof-of-concept trial; ITT preferred, PP exploratory |
| Van den Berg et al., 2016 | Article results text | Quality of life/HRQoL or SIS domain | SIS Strength, 12 weeks, ITT | Aligned adjusted mean difference | -8.2 | -15.5 to -0.8 | Positive values favor the remote intervention package where an aligned effect was derived. | Sensitivity/narrative: proof-of-concept trial; ITT preferred, PP exploratory |
| Whiston et al., 2026 | Article Table 5 | ADL/disability/participation | RNLI, 3-month follow-up | Group-specific adjusted marginal means | EX: 80.11 (95% CI 71.54 to 88.67); LPA: 77.93 (95% CI 69.85 to 86.01) | See result | Not applicable: source-reported group-specific adjusted marginal means. | Active-vs-active first-stage EX versus LPA comparison. The source reported no significant differences or trends for secondary outcomes at post-intervention or 3-month follow-up. A covariance-adjusted between-group contrast and standard error were not reported, so no difference confidence interval was reconstructed. |
| Whiston et al., 2026 | Article Table 5 | ADL/disability/participation | RNLI, postintervention | Group-specific adjusted marginal means | EX: 76.76 (95% CI 66.57 to 86.96); LPA: 78.13 (95% CI 71.11 to 85.16) | See result | Not applicable: source-reported group-specific adjusted marginal means. | Active-vs-active first-stage EX versus LPA comparison. The source reported no significant differences or trends for secondary outcomes at post-intervention or 3-month follow-up. A covariance-adjusted between-group contrast and standard error were not reported, so no difference confidence interval was reconstructed. |
| Whiston et al., 2026 | Article Table 5 | Adherence/feasibility | Adherence %, total intervention | Group-specific adjusted marginal means | EX: 79.24% (95% CI 72.20 to 86.30); LPA: 85.88% (95% CI 77.92 to 93.83) | See result | Not applicable: source-reported group-specific adjusted marginal means. | Active-vs-active first-stage EX versus LPA comparison. The source reported no significant first-stage adherence difference. A covariance-adjusted between-group contrast and standard error were not reported, so no difference confidence interval was reconstructed. |
| Whiston et al., 2026 | Article Table 5 | Depression/anxiety/psychological symptoms | HADS total, 3-month follow-up | Group-specific adjusted marginal means | EX: 9.88 (95% CI 7.08 to 12.69); LPA: 12.33 (95% CI 8.90 to 15.77) | See result | Not applicable: source-reported group-specific adjusted marginal means. | Active-vs-active first-stage EX versus LPA comparison. The source reported no significant differences or trends for secondary outcomes at post-intervention or 3-month follow-up. A covariance-adjusted between-group contrast and standard error were not reported, so no difference confidence interval was reconstructed. |
| Whiston et al., 2026 | Article Table 5 | Depression/anxiety/psychological symptoms | HADS total, postintervention | Group-specific adjusted marginal means | EX: 10.56 (95% CI 7.83 to 13.29); LPA: 14.00 (95% CI 11.13 to 16.87) | See result | Not applicable: source-reported group-specific adjusted marginal means. | Active-vs-active first-stage EX versus LPA comparison. The source reported no significant differences or trends for secondary outcomes at post-intervention or 3-month follow-up. A covariance-adjusted between-group contrast and standard error were not reported, so no difference confidence interval was reconstructed. |
| Whiston et al., 2026 | Article Table 5 | Fatigue | FSS fatigue severity, 3-month follow-up | Group-specific adjusted marginal means | EX: 36.24 (95% CI 31.18 to 41.30); LPA: 36.21 (95% CI 29.11 to 43.31) | See result | Not applicable: source-reported group-specific adjusted marginal means. | Active-vs-active first-stage EX versus LPA comparison. The source reported no significant differences or trends for secondary outcomes at post-intervention or 3-month follow-up. A covariance-adjusted between-group contrast and standard error were not reported, so no difference confidence interval was reconstructed. |
| Whiston et al., 2026 | Article Table 5 | Fatigue | FSS fatigue severity, postintervention | Group-specific adjusted marginal means | EX: 35.08 (95% CI 29.53 to 40.63); LPA: 38.65 (95% CI 29.53 to 40.63) | See result | Not applicable: source-reported group-specific adjusted marginal means. | Active-vs-active first-stage EX versus LPA comparison. The source reported no significant differences or trends for secondary outcomes at post-intervention or 3-month follow-up. A covariance-adjusted between-group contrast and standard error were not reported, so no difference confidence interval was reconstructed. |
| Whiston et al., 2026 | Article Table 5 | Motor function/physical activity | Mean steps/day, 3-month follow-up | Group-specific adjusted marginal means | EX: 8818 (95% CI 7284 to 10351); LPA: 9264 (95% CI 7246 to 11281) | See result | Not applicable: source-reported group-specific adjusted marginal means. | Active-vs-active first-stage EX versus LPA comparison. The source reported no significant differences or trends for secondary outcomes at post-intervention or 3-month follow-up. A covariance-adjusted between-group contrast and standard error were not reported, so no difference confidence interval was reconstructed. |
| Whiston et al., 2026 | Article Table 5 | Motor function/physical activity | Mean steps/day, postintervention | Group-specific adjusted marginal means | EX: 6645 (95% CI 5320 to 7970); LPA: 8529 (95% CI 6646 to 10538) | See result | Not applicable: source-reported group-specific adjusted marginal means. | Active-vs-active first-stage EX versus LPA comparison. The source reported no significant differences or trends for secondary outcomes at post-intervention or 3-month follow-up. A covariance-adjusted between-group contrast and standard error were not reported, so no difference confidence interval was reconstructed. |
| Whiston et al., 2026 | Article Table 5 | Quality of life/HRQoL or SIS domain | EQ-5D VAS, 3-month follow-up | Group-specific adjusted marginal means | EX: 75.28 (95% CI 67.44 to 83.12); LPA: 74.94 (95% CI 64.84 to 85.05) | See result | Not applicable: source-reported group-specific adjusted marginal means. | Active-vs-active first-stage EX versus LPA comparison. The source reported no significant differences or trends for secondary outcomes at post-intervention or 3-month follow-up. A covariance-adjusted between-group contrast and standard error were not reported, so no difference confidence interval was reconstructed. |
| Whiston et al., 2026 | Article Table 5 | Quality of life/HRQoL or SIS domain | EQ-5D VAS, postintervention | Group-specific adjusted marginal means | EX: 80.12 (95% CI 73.15 to 87.09); LPA: 78.11 (95% CI 69.86 to 86.36) | See result | Not applicable: source-reported group-specific adjusted marginal means. | Active-vs-active first-stage EX versus LPA comparison. The source reported no significant differences or trends for secondary outcomes at post-intervention or 3-month follow-up. A covariance-adjusted between-group contrast and standard error were not reported, so no difference confidence interval was reconstructed. |
| Whiston et al., 2026 | Article Table 5 | Quality of life/HRQoL or SIS domain | SS-QOL, 3-month follow-up | Group-specific adjusted marginal means | EX: 194.56 (95% CI 180.44 to 208.68); LPA: 177.94 (95% CI 161.00 to 194.89) | See result | Not applicable: source-reported group-specific adjusted marginal means. | Active-vs-active first-stage EX versus LPA comparison. The source reported no significant differences or trends for secondary outcomes at post-intervention or 3-month follow-up. A covariance-adjusted between-group contrast and standard error were not reported, so no difference confidence interval was reconstructed. |
| Whiston et al., 2026 | Article Table 5 | Quality of life/HRQoL or SIS domain | SS-QOL, postintervention | Group-specific adjusted marginal means | EX: 193.44 (95% CI 178.41 to 204.47); LPA: 172.78 (95% CI 160.14 to 185.42) | See result | Not applicable: source-reported group-specific adjusted marginal means. | Active-vs-active first-stage EX versus LPA comparison. The source reported no significant differences or trends for secondary outcomes at post-intervention or 3-month follow-up. A covariance-adjusted between-group contrast and standard error were not reported, so no difference confidence interval was reconstructed. |

Result/reporting type specifies whether the source provided a mean difference, standardized effect, adjusted coefficient, within-group change with an interaction test, group-specific adjusted marginal means, median contrast, or risk ratio. Positive values favor the remote package only where an aligned effect was defensibly derived. For Linder et al. and Whiston et al., covariance-adjusted between-group contrasts were not reported; no between-group confidence interval was reconstructed. Source locations reproduce the existing extraction record; participant characteristics are provided in Table 2 and the accompanying data file. NR, confidence interval not reported; NA, not applicable; See result, the source-reported group-specific interval is displayed with the result, not a between-group interval. For risk ratios, 1 is the null value. I/C, intervention/control; HEP, home exercise program; EX, structured online exercise; LPA, lifestyle physical activity; ITT, intention-to-treat; PP, per-protocol; MD, mean difference; RR, risk ratio; VAS, visual analogue scale. The Whiston post-intervention SS-QOL EX interval (193.44; 95% CI 178.41 to 204.47) and FSS LPA interval (38.65; 95% CI 29.53 to 40.63) reproduce the published Table 5, including its asymmetric/repeated limits; they were not repaired by assuming symmetry or used to reconstruct between-group effects.

# Table S3. Risk-of-bias details for one representative review-relevant result per trial.

| **Study** | **Target outcome/time point** | **D1 randomization** | **D2 deviations** | **D3 missing outcome data** | **D4 outcome measurement** | **D5 selective reporting** | **Overall RoB 2** | **Main reasons** | **Synthesis handling** |
| --- | --- | --- | --- | --- | --- | --- | --- | --- | --- |
| Fusari et al., 2025 | EQ-5D-5L at 12 weeks | Low risk | Some concerns | Some concerns | Some concerns | Some concerns | Some concerns | Exploratory self-reported outcome; missing data and unavailable prespecified outcome analysis. | Evidence map and descriptive/sensitivity synthesis. |
| Grau-Pellicer et al., 2020 | EQ-5D-5L problem score at 3 months | Some concerns | Some concerns | Some concerns | High risk | Some concerns | High risk | Small unblinded pilot with subjective measurement and reporting concerns. | Exploratory standardized display only. |
| Linder et al., 2015 | CES-D change at end of treatment | Some concerns | Some concerns | Low risk | Some concerns | Some concerns | Some concerns | Active-vs-active comparison; incomplete allocation and prespecified-reporting information. | Source-reported within-group changes and time x group interaction; no reconstructed between-group confidence interval. |
| Sun et al., 2025 | HAMD at 12 weeks | Low risk | Low risk | Low risk | Low risk | Some concerns | Some concerns | Random number table, sealed opaque envelopes, blinded assessors, and ITT were reported; analysis plan unavailable. | Sole direct depression effect; package-level sensitivity interpretation. |
| van den Berg et al., 2016 | HADS at 12 weeks | Low risk | Some concerns | Some concerns | Some concerns | Some concerns | Some concerns | Proof-of-concept trial; the representative result was the patient SIS Memory domain at 12 weeks under the ITT analysis. Missing data and incomplete prespecified-reporting information created some concerns. The caregiver HADS result was not the target of this RoB 2 assessment. | Patient SIS evidence retained with cautious interpretation; caregiver HADS findings were not used as patient psychiatric effects. |
| Whiston et al., 2026 | HADS total post-intervention | Low risk | Some concerns | Some concerns | Some concerns | Some concerns | Some concerns | Feasibility SMART with missing data and model-based exploratory reporting. | Group-specific adjusted marginal means and source-reported first-stage inference retained narratively; no reconstructed difference confidence interval. |

Domain judgments follow the Cochrane RoB 2 framework: D1 randomization process, D2 deviations from intended interventions, D3 missing outcome data, D4 outcome measurement, and D5 selection of the reported result. These judgments are not assumed to apply to all outcomes reported by the same trial.

# Table S4. Descriptive evidence sets and psychological-support sensitivity analysis.

| **Set** | **Purpose** | **Study ID** | **Study** | **Retained** | **Interpretation** |
| --- | --- | --- | --- | --- | --- |
| S0 | All six included randomized studies: evidence mapping and descriptive synthesis. | Fusari2025 | Fusari et al., 2025 | Yes | Retained for evidence mapping and descriptive synthesis. |
| S0 | All six included randomized studies: evidence mapping and descriptive synthesis. | GrauPellicer2020 | Grau-Pellicer et al., 2020 | Yes | Retained for evidence mapping and descriptive synthesis. |
| S0 | All six included randomized studies: evidence mapping and descriptive synthesis. | Linder2015 | Linder et al., 2015 | Yes | Retained for evidence mapping and descriptive synthesis. |
| S0 | All six included randomized studies: evidence mapping and descriptive synthesis. | Sun2025 | Sun et al., 2025 | Yes | Retained for evidence mapping and descriptive synthesis. |
| S0 | All six included randomized studies: evidence mapping and descriptive synthesis. | VanDenBerg2016 | van den Berg et al., 2016 | Yes | Retained for evidence mapping and descriptive synthesis. |
| S0 | All six included randomized studies: evidence mapping and descriptive synthesis. | Whiston2026 | Whiston et al., 2026 | Yes | Retained for evidence mapping and descriptive synthesis. |
| S1 | Descriptive subset with a directly analyzable standalone depression contrast against usual or standard care. | Fusari2025 | Fusari et al., 2025 | No | No standalone depression effect; exploratory QoL data remain in the full evidence map. |
| S1 | Descriptive subset with a directly analyzable standalone depression contrast against usual or standard care. | GrauPellicer2020 | Grau-Pellicer et al., 2020 | No | No standalone depression effect; the comparator is active conventional rehabilitation. |
| S1 | Descriptive subset with a directly analyzable standalone depression contrast against usual or standard care. | Linder2015 | Linder et al., 2015 | No | Active remote comparator; CES-D within-group changes and the interaction test are retained separately. |
| S1 | Descriptive subset with a directly analyzable standalone depression contrast against usual or standard care. | Sun2025 | Sun et al., 2025 | Yes | Standalone HAMD contrast against standard care is analyzable; psychological counseling limits attribution. |
| S1 | Descriptive subset with a directly analyzable standalone depression contrast against usual or standard care. | VanDenBerg2016 | van den Berg et al., 2016 | No | No separable patient depression estimate in the available extraction; caregiver results are not patient effects. |
| S1 | Descriptive subset with a directly analyzable standalone depression contrast against usual or standard care. | Whiston2026 | Whiston et al., 2026 | No | HADS total is combined distress and both strategies are active remote interventions. |
| S2 | Descriptive set adding activity-use and mixed mHealth QoL studies to S1; comparator differences retained. | Fusari2025 | Fusari et al., 2025 | Yes | Retained in this descriptive subset; not a pooled sensitivity estimate. |
| S2 | Descriptive set adding activity-use and mixed mHealth QoL studies to S1; comparator differences retained. | GrauPellicer2020 | Grau-Pellicer et al., 2020 | Yes | Retained in this descriptive subset; not a pooled sensitivity estimate. |
| S2 | Descriptive set adding activity-use and mixed mHealth QoL studies to S1; comparator differences retained. | Linder2015 | Linder et al., 2015 | No | Outside this descriptive subset; remains in the full six-study evidence map. |
| S2 | Descriptive set adding activity-use and mixed mHealth QoL studies to S1; comparator differences retained. | Sun2025 | Sun et al., 2025 | Yes | Retained in this descriptive subset; not a pooled sensitivity estimate. |
| S2 | Descriptive set adding activity-use and mixed mHealth QoL studies to S1; comparator differences retained. | VanDenBerg2016 | van den Berg et al., 2016 | No | Outside this descriptive subset; remains in the full six-study evidence map. |
| S2 | Descriptive set adding activity-use and mixed mHealth QoL studies to S1; comparator differences retained. | Whiston2026 | Whiston et al., 2026 | No | Outside this descriptive subset; remains in the full six-study evidence map. |
| S3 | Extended descriptive set including active conventional and active remote comparisons. | Fusari2025 | Fusari et al., 2025 | Yes | Retained with comparator-specific interpretation; no pooled estimate. |
| S3 | Extended descriptive set including active conventional and active remote comparisons. | GrauPellicer2020 | Grau-Pellicer et al., 2020 | Yes | Retained with comparator-specific interpretation; no pooled estimate. |
| S3 | Extended descriptive set including active conventional and active remote comparisons. | Linder2015 | Linder et al., 2015 | Yes | Retained with comparator-specific interpretation; no pooled estimate. |
| S3 | Extended descriptive set including active conventional and active remote comparisons. | Sun2025 | Sun et al., 2025 | Yes | Retained with comparator-specific interpretation; no pooled estimate. |
| S3 | Extended descriptive set including active conventional and active remote comparisons. | VanDenBerg2016 | van den Berg et al., 2016 | Yes | Retained with comparator-specific interpretation; no pooled estimate. |
| S3 | Extended descriptive set including active conventional and active remote comparisons. | Whiston2026 | Whiston et al., 2026 | Yes | Retained with comparator-specific interpretation; no pooled estimate. |
| S4 | Full evidence map distinguishing global QoL measures from individual SIS domains. | Fusari2025 | Fusari et al., 2025 | Yes | Retained; SIS domain findings are not interpreted as global QoL effects. |
| S4 | Full evidence map distinguishing global QoL measures from individual SIS domains. | GrauPellicer2020 | Grau-Pellicer et al., 2020 | Yes | Retained; SIS domain findings are not interpreted as global QoL effects. |
| S4 | Full evidence map distinguishing global QoL measures from individual SIS domains. | Linder2015 | Linder et al., 2015 | Yes | Retained; SIS domain findings are not interpreted as global QoL effects. |
| S4 | Full evidence map distinguishing global QoL measures from individual SIS domains. | Sun2025 | Sun et al., 2025 | Yes | Retained; SIS domain findings are not interpreted as global QoL effects. |
| S4 | Full evidence map distinguishing global QoL measures from individual SIS domains. | VanDenBerg2016 | van den Berg et al., 2016 | Yes | Retained; SIS domain findings are not interpreted as global QoL effects. |
| S4 | Full evidence map distinguishing global QoL measures from individual SIS domains. | Whiston2026 | Whiston et al., 2026 | Yes | Retained; SIS domain findings are not interpreted as global QoL effects. |
| S5 | Sensitivity analysis excluding an explicit psychological-support co-intervention. | Fusari2025 | Fusari et al., 2025 | Yes | Retained, but this study does not provide a directly analyzable depression contrast against standard care. |
| S5 | Sensitivity analysis excluding an explicit psychological-support co-intervention. | GrauPellicer2020 | Grau-Pellicer et al., 2020 | Yes | Retained, but this study does not provide a directly analyzable depression contrast against standard care. |
| S5 | Sensitivity analysis excluding an explicit psychological-support co-intervention. | Linder2015 | Linder et al., 2015 | Yes | Retained, but this study does not provide a directly analyzable depression contrast against standard care. |
| S5 | Sensitivity analysis excluding an explicit psychological-support co-intervention. | Sun2025 | Sun et al., 2025 | No | Excluded because the intervention explicitly included mental-health education and online psychological counseling; its removal leaves no direct analyzable depression contrast against standard care. |
| S5 | Sensitivity analysis excluding an explicit psychological-support co-intervention. | VanDenBerg2016 | van den Berg et al., 2016 | Yes | Retained, but this study does not provide a directly analyzable depression contrast against standard care. |
| S5 | Sensitivity analysis excluding an explicit psychological-support co-intervention. | Whiston2026 | Whiston et al., 2026 | Yes | Retained, but this study does not provide a directly analyzable depression contrast against standard care. |

The S5 scenario excludes the Sun et al. trial because it explicitly included psychological counseling. No pooled depression estimate was available to recalculate; after exclusion, no direct analyzable depression contrast against standard care remained. S0-S4 organize descriptive evidence; they are not additional eligibility criteria or recalculated meta-analyses. All six studies remain included regardless of whether the relevant outcome was primary or secondary in the source trial. The S5 result concerns the available depression evidence set, not a new pooled effect.

# Table S5. Potential heterogeneity variables for descriptive comparison.

| **Study ID** | **Study** | **Remote delivery** | **Technology** | **Comparator** | **Active content** | **Weeks** | **N** | **Pilot** | **SMART** | **Active control** | **Psych. support** | **QoL construct** | **Mood measure** | **Data format** | **RoB 2** | **Synthesis role** | **Comparator class** |
| --- | --- | --- | --- | --- | --- | --- | --- | --- | --- | --- | --- | --- | --- | --- | --- | --- | --- |
| Fusari2025 | Fusari et al., 2025 | Wearable/digital feedback plus coaching | Wearable/app feedback | Site-specific NHS multidisciplinary rehabilitation; not standardized | Activity-use promotion; no fixed exercise prescription | 12 | 30 | Yes | No | Yes | No | EQ-5D-5L | No | No | Some concerns | Descriptive QoL evidence | Routine NHS multidisciplinary rehabilitation; active and variable |
| GrauPellicer2020 | Grau-Pellicer et al., 2020 | mHealth, pedometer, WhatsApp plus exercise sessions | mHealth/app/WhatsApp | Conventional rehabilitation | Multimodal PA/walking/exercise programme | 8 | 41 | Yes | No | Yes | No | EQ-5D problem score (not utility) | No | Yes for selected outcomes | High | Exploratory active-comparator effects | Active conventional rehabilitation |
| Linder2015 | Linder et al., 2015 | Robot-assisted home training plus telephone monitoring | Robot/device plus telephone | Active remote-supervised HEP | Fixed upper-limb home programme | 8 | 99 | No | No | Yes | No | SIS domains | CES-D | No; source interaction test used | Some concerns | Active remote comparison; narrative | Active remote-supervised comparator |
| Sun2025 | Sun et al., 2025 | Telerehabilitation platform plus weekly video consultation | Platform/video/games | Standard post-discharge care | Multidisciplinary telerehabilitation including PT/OT/speech | 12 | 200 | No | No | No | Yes - mental-health education and online psychological counseling | WHOQOL-BREF | HAMD | HAMD and BI yes; WHOQOL beta only | Some concerns | Direct depression contrast; psychological-support sensitivity | Minimal/usual/wait-list or standard care |
| VanDenBerg2016 | van den Berg et al., 2016 | Caregiver-mediated exercise with app/videoconference/Fitbit | App/video/wearable | Conventional multidisciplinary rehabilitation | Caregiver-mediated walking/function exercises | 8 | 63 | Proof-of-concept | No | Yes | No | SIS domains | Patient HADS collected; separable patient effect unavailable in main report | No; adjusted narrative results | Some concerns | Active conventional comparison; narrative | Active conventional rehabilitation |
| Whiston2026 | Whiston et al., 2026 | Adaptive mHealth PA intervention with Fitbit | App/wearable/text/video | Active remote PA strategy | EX: structured online exercise; LPA: lifestyle activity and behavior change | 12 | 50 | Yes | Yes | Yes | No | SS-QOL and EQ-5D VAS | HADS total | No; group-specific adjusted marginal means | Some concerns | Active remote SMART; narrative | Active remote-supervised comparator |

These variables describe potential sources of heterogeneity and retain the information available for candidate subgroup or meta-regression analyses. No formal subgroup meta-analysis or meta-regression was conducted because comparable study counts were insufficient. Pilot includes feasibility and proof-of-concept designs; Psych. support denotes an explicit psychological co-intervention. QoL construct distinguishes global measures from SIS domains; the psychiatric measure column distinguishes standalone scales, combined distress, and unavailable patient estimates. N is the randomized denominator. PA, physical activity; HEP, home exercise program; EX, structured online exercise; LPA, lifestyle physical activity; BI, Barthel Index; SIS, Stroke Impact Scale; HADS, Hospital Anxiety and Depression Scale; HAMD, Hamilton Depression Rating Scale; CES-D, Center for Epidemiologic Studies Depression Scale.

# Table S6. GRADE certainty of evidence.

| **Outcome/evidence body** | **Included studies** | **Number of studies** | **Randomized N** | **Findings by evidence domain** | **Risk of bias** | **Inconsistency** | **Indirectness** | **Imprecision** | **Publication bias** | **Certainty** | **Main reasons** |
| --- | --- | --- | --- | --- | --- | --- | --- | --- | --- | --- | --- |
| Depression or psychological distress | Linder 2015; Sun 2025; Whiston 2026 | 3 | 349 randomized participants | Sun 2025 provided the only direct standard-care depression effect and favored the multicomponent package. In Linder 2015, both groups improved in CES-D but the time x group interaction was not significant (p=0.553). Whiston 2026 reported no significant first-stage EX-versus-LPA differences or trends for secondary outcomes. | Serious | Serious | Serious | Serious | Not assessable | Very low | Direct evidence depends on one trial with explicit psychological counseling; the remaining studies used active comparators and did not provide covariance-adjusted between-group contrasts suitable for confidence-interval reconstruction. |
| Anxiety | Whiston 2026; van den Berg 2016: combined HADS or patient HADS collection | 0 separable anxiety effects (2 reports with related measurement) | 113 randomized in those reports; no anxiety-effect denominator | No separable patient anxiety effect was available. HADS total cannot identify an anxiety-subscale treatment effect. | Not assessed | Not assessed | Not assessed | Not assessed | Not assessed | Not rated | Outcome availability gap, not indirect treatment evidence. No GRADE category assigned in the absence of an interpretable anxiety-specific effect. |
| Quality of life or health-related quality of life | Fusari 2025; Grau-Pellicer 2020; Linder 2015; Sun 2025; van den Berg 2016; Whiston 2026 | 6 | 483 randomized participants | QoL-related outcomes were reported, but instruments and contrasts varied substantially. | Serious | Serious | Serious | Serious | Not assessable | Very low | Different instruments, mixed global/domain scales, small samples and active or feasibility designs. |
| Activities of daily living | Grau-Pellicer 2020; Sun 2025; van den Berg 2016 | 3 | 304 randomized participants | Some favorable effects were observed for Barthel Index, particularly in Sun 2025. | Serious | Serious | Serious | Serious | Not assessable | Very low | Heterogeneous settings and interventions; limited number of studies. |
| Motor or physical function | Grau-Pellicer 2020; Fusari 2025; Linder 2015; Sun 2025; van den Berg 2016; Whiston 2026 | 6 | 483 randomized participants | Several outcomes favored remote or digitally supported rehabilitation, but measures were highly heterogeneous. | Serious | Serious | Serious | Serious | Not assessable | Very low | Outcome diversity and feasibility/active-comparator designs limit certainty. |
| Safety and adherence | Fusari 2025; Grau-Pellicer 2020; Linder 2015; Sun 2025; van den Berg 2016; Whiston 2026 | 6 | 483 randomized participants | Interventions were generally feasible, and serious adverse events were uncommon or not attributed to the intervention where reported. | Serious | Serious | Serious | Serious | Not assessable | Very low | Safety and adherence definitions varied and samples were underpowered for uncommon harms. |

GRADE categories were assigned to outcome-level evidence bodies with interpretable results after considering risk of bias, inconsistency, indirectness, imprecision, and publication bias. Anxiety is shown solely as an outcome-availability row and was not rated because no separable patient anxiety effect was available. The 113 participants identify the two reports with HADS-related information, not an analyzable anxiety sample. Other sample sizes are descriptive randomized totals rather than pooled outcome-specific denominators; participants and outcome domains overlap. Comparator classes are not interpreted as a common treatment effect.

# Table S7. Baseline psychiatric eligibility and symptom characteristics, and psychological or behavioral co-interventions.

| **Study** | **Psychiatric eligibility/recruitment** | **Baseline psychiatric characteristics** | **Psychological/behavioral co-interventions** | **Interpretive implication** |
| --- | --- | --- | --- | --- |
| Fusari et al., 2025 | No depression or anxiety eligibility criterion; participants were recruited for upper-limb rehabilitation. | No symptom-enrichment strategy; baseline depression/anxiety distribution was not reported as a recruitment characteristic. | Self-management coaching and therapist feedback; no explicit formal psychological treatment reported. | QoL was exploratory; no standalone depression or anxiety outcome was reported. |
| Grau-Pellicer et al., 2020 | No depression or anxiety eligibility criterion; chronic ambulatory stroke sample. | No symptom-enrichment strategy or baseline psychiatric case definition reported. | Bidirectional WhatsApp feedback, monitoring, and supervised rehabilitation; no explicit psychological counseling. | QoL/functional effects cannot be interpreted as treatment effects in a psychiatric sample. |
| Linder et al., 2015 | No depression eligibility criterion; recruited for upper-limb telerehabilitation and limited access to formal rehabilitation. | Among completers, CES-D >16 was reported in 27.5% of HEP and 26.7% of robot+HEP participants. | Weekly telephone monitoring and substantial home-practice contact in both active arms; no formal psychotherapy. | Active-vs-active design estimates incremental modality effects, not exercise versus no treatment. |
| Sun et al., 2025 | No confirmed post-stroke depression diagnosis required; acute ischemic stroke sample recruited after discharge. | Mean baseline HAMD was 17.5 (SD 4.2) in intervention and 17.3 (SD 4.0) in control. | Mental-health education and online psychological counseling were explicit intervention components. | The sole direct depression signal is package-level and cannot be attributed to exercise alone. |
| van den Berg et al., 2016 | Initially required patient HADS depression score <11; HADS screening was removed after 5 months because acute anxiety restricted recruitment. | Patient and caregiver HADS measures were collected, but no symptom-enriched psychiatric sample was recruited. | Caregiver-mediated practice, weekly evaluation, videoconferencing, app, and wearable feedback; no formal psychological treatment. | The accessible main report did not provide a separable patient anxiety effect; the 2.9-point per-protocol estimate appeared in caregiver-burden results and was not used as a patient effect. |
| Whiston et al., 2026 | No depression or anxiety eligibility criterion; community stroke survivors recruited for physical-activity intervention development. | Baseline HADS total model means were 11.59 for EX and 15.19 for LPA; no psychiatric diagnosis was required. | Therapist coaching, Fitbit feedback, behavior-change support, and secure remote contact in both active strategies. | HADS total is a combined distress measure. The source reported no significant first-stage differences or trends for secondary outcomes; these feasibility findings are not confirmatory psychiatric effects. |

The table distinguishes psychiatric sample enrichment from intervention components that could confound attribution of mental-health effects to exercise alone.

# Table S8. Machine-readable evidence-map data for digital accessibility.

| **Study** | **Depression/distress** | **Anxiety** | **QoL/HRQoL** | **ADL/disability** | **Motor/physical activity** | **Safety/adherence** |
| --- | --- | --- | --- | --- | --- | --- |
| Fusari et al., 2025 | Not reported | Not reported | Reported | Reported | Reported | Reported |
| Grau-Pellicer et al., 2020 | Not reported | Not reported | Reported | Reported | Reported | Reported |
| Linder et al., 2015 | Direct | Not reported | Reported | Reported | Reported | Reported |
| Sun et al., 2025 | Direct | Not reported | Reported | Reported | Reported | Reported |
| van den Berg et al., 2016 | Collected; no separable patient estimate | Collected; no separable patient estimate | Reported | Reported | Reported | Reported |
| Whiston et al., 2026 | Combined | Combined | Reported | Reported | Reported | Reported |

Direct indicates a standalone depression or anxiety measure; Combined indicates a non-separable patient distress measure; Collected; no separable patient estimate indicates that the construct was measured but no separable patient estimate was available; Reported indicates that a non-psychiatric outcome domain was reported; and Not reported indicates absence of eligible data. The table corresponds exactly to Figure 4 and does not encode effect direction, magnitude, or certainty.

# Supplementary Results S1. Contextual functional, activity, adherence, and safety outcomes.

Activities of daily living and disability were reported with the Barthel Index, modified Rankin Scale, NEADL, RNLI, or related constructs. Exploratory aligned standardized effects were conventionally large for the Barthel Index in Grau-Pellicer et al. (g 0.94, 95% CI 0.21 to 1.67) and Sun et al. (g 1.19, 95% CI 0.89 to 1.49); Sun et al. also reported an mRS 0-2 risk ratio of 1.21 (95% CI 1.00 to 1.46). van den Berg et al. reported favorable per-protocol NEADL contrasts but no clear superiority for the primary intention-to-treat mobility outcome. In the small Grau-Pellicer pilot, aligned effects for walking, mobility, and physical activity ranged from g 0.69 to 1.22; motor and activity outcomes in the other trials were too heterogeneous for a common estimate. Reported adherence included 90.7% median weekly OnTrack use in Fusari et al., 82.2% total-intervention adherence in Whiston et al., and approximately 50% app compliance in Grau-Pellicer et al.; definitions were not harmonized. Serious adverse events were uncommon and generally not attributed to the intervention where reported, but samples were too small to exclude uncommon harms. Functional gains could plausibly support mental health through greater autonomy, self-efficacy, and participation, but none of the trials tested these outcomes as mediators.

These secondary results were not combined with depression, anxiety, or global quality-of-life outcomes. Favorable functional findings do not demonstrate psychiatric efficacy or mediation. Complete extracted results and source locations are retained in Table S2; standardized functional/activity contrasts are shown in Supplementary Figure S1.

# Supplementary Figure S1. Exploratory standardized functional and activity effects.

Study-specific Hedges g values for activities of daily living, walking, mobility, and activity/sedentary outcomes. Positive values favor the remote intervention package; no pooled estimate is presented. The original point estimates and standard errors are retained. These are contextual rehabilitation outcomes, not substitutes for depression, anxiety, or quality of life. Conventional standardized-effect reference values do not indicate clinical importance. The Grau-Pellicer outcomes were assessed at 3 months and the Sun Barthel Index at 12 weeks. 10MWT, 10-meter walk test; CI, confidence interval.
